# Systematic discovery of recombinases for efficient integration of large DNA sequences into the human genome

**DOI:** 10.1038/s41587-022-01494-w

**Published:** 2022-10-10

**Authors:** Matthew G. Durrant, Alison Fanton, Josh Tycko, Michaela Hinks, Sita S. Chandrasekaran, Nicholas T. Perry, Julia Schaepe, Peter P. Du, Peter Lotfy, Michael C. Bassik, Lacramioara Bintu, Ami S. Bhatt, Patrick D. Hsu

**Affiliations:** 1 Arc Institute, Palo Alto, CA, USA.; 2 Department of Bioengineering, University of California, Berkeley, Berkeley, CA, USA; 3 Department of Genetics, Stanford University, Stanford, CA, USA; 4 University of California, Berkeley—University of California, San Francisco Graduate Program in Bioengineering, Berkeley, CA, USA; 5 Department of Bioengineering, Stanford University, Stanford, CA, USA; 6 Cancer Biology Program, Stanford University, Stanford, CA, USA; 7 Laboratory of Molecular and Cell Biology, Salk Institute for Biological Studies, La Jolla, CA, USA; 8 Department of Medicine (Hematology), Stanford University, Stanford, CA, USA; 9 Innovative Genomics Institute, University of California, Berkeley, Berkeley, CA, USA; 10 Center for Computational Biology, University of California, Berkeley, Berkeley, CA, USA; 11 These authors contributed equally: Matthew G. Durrant, Alison Fanton, Josh Tycko

## Abstract

Large serine recombinases (LSRs) are DNA integrases that facilitate the site-specific integration of mobile genetic elements into bacterial genomes. Only a few LSRs, such as Bxb1 and PhiC31, have been characterized to date, with limited efficiency as tools for DNA integration in human cells. In this study, we developed a computational approach to identify thousands of LSRs and their DNA attachment sites, expanding known LSR diversity by >100-fold and enabling the prediction of their insertion site specificities. We tested their recombination activity in human cells, classifying them as landing pad, genome-targeting or multi-targeting LSRs. Overall, we achieved up to seven-fold higher recombination than Bxb1 and genome integration efficiencies of 40–75% with cargo sizes over 7 kb. We also demonstrate virus-free, direct integration of plasmid or amplicon libraries for improved functional genomics applications. This systematic discovery of recombinases directly from microbial sequencing data provides a resource of over 60 LSRs experimentally characterized in human cells for large-payload genome insertion without exposed DNA double-stranded breaks.

The ability to clone, modify and edit DNA molecules largely relies on effectors derived from microbial or bacteriophage enzymes governing phage–bacterial warfare^1,2^. Manipulation of eukaryotic genomes, particularly the integration of multi-kilobase (kb) DNA sequences, remains challenging and limits the rapidly growing fields of synthetic biology, cell engineering and gene therapy. Current gene integration approaches primarily rely on nuclease-directed DNA double-stranded breaks (DSBs) to direct cellular DNA repair pathways, such as homologous recombination (HR). Despite important advances in optimizing HR in specific contexts^3,4^, these approaches generally suffer from low insertion efficiency, high indel rates and cargo size limitations, with limited success for cargoes larger than 1 kb^5–7^. Furthermore, HR-based gene editing is not feasible in post-mitotic cells, and formation of DSBs is toxic in many primary cell types, leading to undesired deletions, complex rearrangements^8^ or activation of p53 (ref. ^9^).

Bacterial and phage integrase systems, such as site-specific recombinases, exhibit natural mechanistic advantages to address these key limitations. These enzymes have evolved to catalyze the transfer of large genetic payloads, such as entire phage genomes or conjugative elements (collectively, mobile genetic elements (MGEs)) that are often tens of kilobases in length, from one organism to another, without relying on recipient genetic repair machinery. By recognizing attachment sites—their recognition sequences found on DNA donor and acceptor molecules—recombinases are capable of catalyzing target cleavage, strand exchange and DNA rejoining within their synaptic complex. This mechanism enables site-specific DNA insertion without requiring any cellular cofactors and without generating exposed DSBs.

Tyrosine recombinases, such as Cre and Flp, are widely used for genome manipulation but require engineering to overcome their inherent reaction bidirectionality that favors re-excision of an integration product^10–13^. In contrast, LSRs, such as Bxb1 and PhiC31, can catalyze unidirectional DNA integration into cognate attachment sites^14,15^. PhiC31 can integrate payloads into pseudosite loci in eukaryotic genomes that resemble its native attachment sites^16,17^, whereas Bxb1 requires pre-installation of its preferred attachment site in the human genome^18^. A major advantage of LSRs over other emerging technologies is that there is no obvious upper limit on the size of the donor DNA, with reports demonstrating successful 27-kb integration into mammalian cells with Bxb1 (ref. ^19^). Although these features make LSRs highly attractive genome editing tools, the practical application of existing LSRs has been limited by several factors, most notably their low integration efficiency that necessitates further experimental enrichment of successful integrants^15,20^.

Past efforts to develop LSR tools have largely focused on enhancing the few known recombinases through processes such as directed evolution, protein fusion, domain swapping and delivery optimization^21–24^. However, the advent of extensive microbial genomics and metagenomics efforts has presented the opportunity to discover millions of new genes^25,26^. We reasoned that the abundance of both sequenced genomes and LSR proteins in nature provides an opportunity to mine natural systems that are directly useful for human genome editing.

In this study, we sought to expand the LSR toolbox by systematic computational identification of LSRs from bacterial MGEs, followed by experimental characterization of their capacity to integrate genetic cargo into the human genome. By developing an approach that enables the prediction of MGE boundaries in a highly precise and automated fashion^27^, we were able to systematically reconstruct the cognate DNA recognition sites for thousands of LSRs at a larger scale relative to previous methods^28^.

Next, we synthesized and functionally tested over 60 diverse LSRs. The most efficient new LSRs vastly outperformed existing recombinases, achieving up to seven-fold higher plasmid recombination than Bxb1 and genome insertion efficiencies of 40–75% with cargo sizes over 7 kb. Taking advantage of these key features, we applied LSRs from our collection to demonstrate three key applications: (1) a new method for amplicon library installation at genomic landing pads; (2) genomic integration of cargos and the integration of multiple constructs in the same cell simultaneously; and (3) direct targeting of specific sites in the human genome with higher efficiency than PhiC31. Altogether, our study emphasizes the untapped potential of integrase enzymes for developing several new classes of tools to manipulate the human genome and overcome the limitations of existing technologies.

## Results

### Systematic discovery of LSRs and their target sites

**Fig. 1 |:**
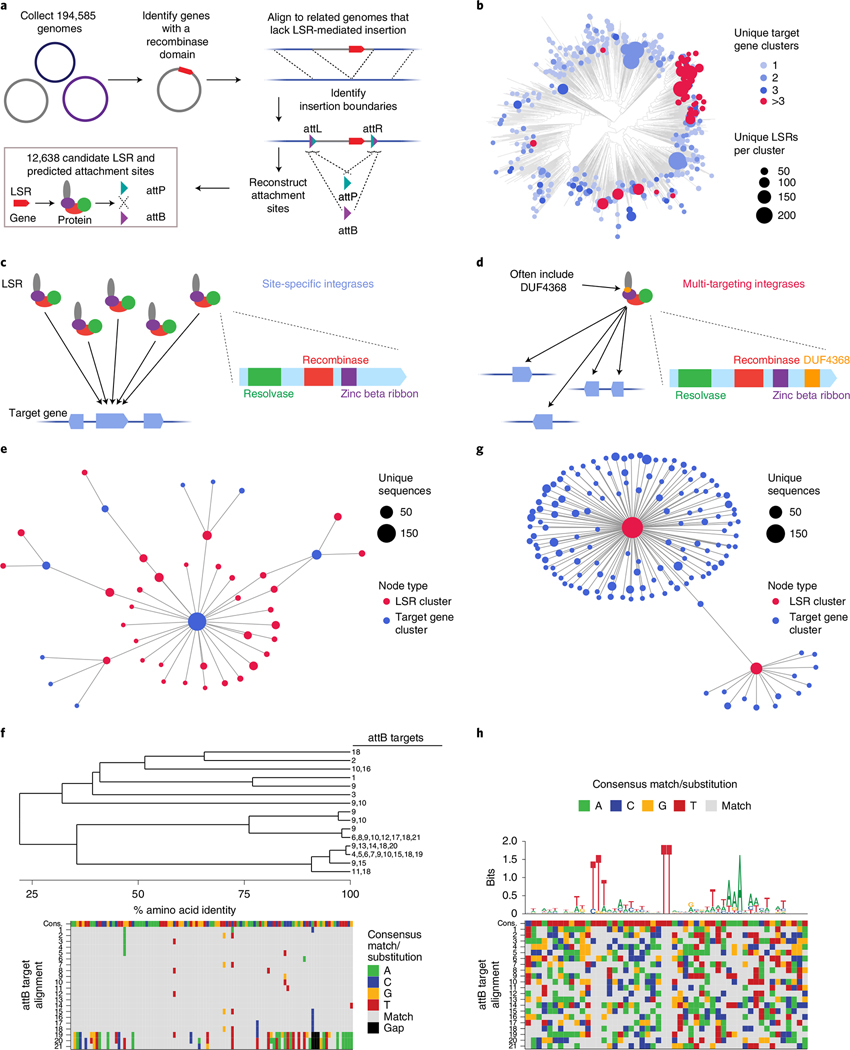
Systematic discovery and classification of LSRs and their target site specificities. **a**, Schematic of the computational workflow for systematic identification of LSRs and inference of their attachment sites. The gene harboring the recombinase domain is shown as a red rectangle. **b**, Phylogenetic tree of representative LSR orthologs clustered at 50% identity, annotated according to predicted target specificity of each LSR cluster. ‘Unique target gene clusters’ indicates the number of predicted target gene clusters, dots scaled to indicate the number of unique LSR sequences found in each LSR cluster. **c**, Schematic of the technique to identify site-specific LSRs that target a single gene cluster. The typical domain architecture of a site-specific LSR is illustrated. **d**, Schematic of the technique to identify multi-targeting LSRs. In brief, if a single cluster of related LSRs (clustered at 90% identity) integrates into multiple diverse target gene clusters (clustered at 50% identity), then the LSR cluster is considered multitargeting. The typical domain architecture of a multi-targeting LSR is illustrated, commonly including a particular domain of unknown function, DUF4368. **e**, Example of an observed network of predicted site-specific LSRs found in our database. Each node indicates either an LSR cluster (red) or a target gene cluster (blue). Edges between nodes indicate that at least one member of the LSR cluster was found integrated into at least one member of the target gene cluster. **f**, Example of a hierarchical tree of diverse LSR sequences that target a set of closely related attB sequences. Numbers at the tip of the tree indicate the attB sequences in the alignment that are targeted by each LSR. Bottom is the alignment of related attB sequences. **g**, Example of an observed network of predicted multitargeting LSRs. **h**, Schematic of an alignment of diverse attB sequences that are targeted by a single multi-targeting LSR. Each target sequence is aligned with respect to the core TT dinucleotide. Sequence logo above the alignment indicates conservation across target sites, a proxy for the sequence specificity of this particular LSR. The alignment is colored according to the consensus.

LSRs canonically recombine two distinct DNA attachment sites natively found on an invading MGE (attP) and in the target bacterial genome (attB). Upon MGE insertion, the attachment sites are retained at the boundaries of the integrated segment, forming attL and attR. We sought to systematically identify LSRs contained within MGEs and their attachment sites using a comparative genomics approach across 194,585 clinical and environmental bacterial isolate genomes (Fig. 1a). By comparing genomes with and without integrants, we identified the boundaries of the integrant and used the attL and attR sequences to reconstruct the original attP and attB attachment sites for 12,638 candidates. After applying quality control filters, such as coding sequence length or LSR distance from their predicted attachment site, our final dataset of LSR attachment site predictions included 6,207 unique LSRs (1,081 50% amino acid identity clusters) and cognate attachment sites (Supplementary Table 1). These candidates belonged to 20 host phyla, indicating good representation of published bacterial assemblies (Supplementary Fig. 1a). Although our approach to LSR identification is agnostic to MGE annotation, we predicted 50.7% of LSR-carrying MGEs to be prophage and 4.8% to be integrative and conjugative elements (ICEs)/integrative and mobilizable elements (IMEs), plasmids or other replicons, and 44.5% could not be classified, demonstrating that our pipeline is more comprehensive relative to previous techniques that have relied on prophage annotations alone^28^.

Next, we sought to bioinformatically predict the site specificity of these LSRs (Fig. 1b). To do this, we compared integration patterns across LSR clusters, grouping attB target sites by the bacterial gene clusters that were disrupted upon MGE insertion, referred to as target gene clusters. We reasoned that if many distantly related LSRs were found to target the same target gene clusters, it is likely that these LSRs would be site-specific (Fig. 1c). We predict that 82.8–88.3% of LSR clusters are relatively site-specific, targeting 1–3 unique target gene clusters. Conversely, if we saw LSR clusters that targeted many distinct target gene clusters, we classified them as ‘multi-targeting’, meaning that they either had relaxed sequence specificity and/or they evolved to target sequences that occurred at multiple different sites in their host organisms (Fig. 1d). One notable clade contains many multi-targeting LSRs that are predicted to integrate into more than three target gene clusters, suggesting that this was an evolved strategy inherited from a common ancestor (Fig. 1b and Supplementary Fig. 1a). We observed that most (63%) LSRs in this clade contain DUF4368, a Pfam domain of unknown function, which is rarely (0.73%) found in site-specific LSRs (Supplementary Fig. 1a), and that the clade includes LSRs in the TndX-like transposase subfamily^29^.

We found many examples of distantly related LSRs that targeted the same gene clusters, including several diverse LSR clusters that primarily target a single gene cluster (Fig. 1e,f). Homologs of this particular gene, annotated as a magnesium chelatase/ATP-dependent protease, are significantly enriched among target genes (Supplementary Fig. 1b) and are targeted by 12.4% of all predicted site-specific integrases. In another striking example, we found a diverse set of 33 unique LSRs (15 99% amino acid identity clusters and six 50% identity clusters) that all target a single conserved site within the coding sequence of a prolyl isomerase (Fig. 1f). A more comprehensive analysis of target genes and their associated gene pathways revealed strong enrichment in DNA competence genes and no enrichment within or near anti-phage defense genes (Supplementary Fig. 1b–d and Supplementary Note 1).

We also identified clusters of LSRs where closely related orthologs integrate into divergent target gene clusters (Fig. 1g). Several of these multi-targeting LSRs have large numbers of associated attB target sites, which allows us to infer their sequence specificity computationally from our database. In one example, we found a single multi-targeting integrase that targets 21 distinct attB sites. An alignment of these target sites revealed a conserved TT dinucleotide core with 5′ and 3′ ends enriched for T and A nucleotides, suggesting that this ortholog most likely has relaxed sequence specificity overall (Fig. 1h). Other multi-targeting LSRs appear to have distinct target site motifs, including several with motifs that are more complex than short, AT-rich sequences (Supplementary Fig. 1e). Overall, these analyses demonstrate the power of large-scale discovery of LSRs and attachment sites, as they provide insight into the differences in targeting specificity across the diversity of serine integrases. Furthermore, they suggest that we may be able to predict the ability of these integrases to target any given genome.

### Development of efficient landing pad LSRs in human cells

**Fig. 2 |:**
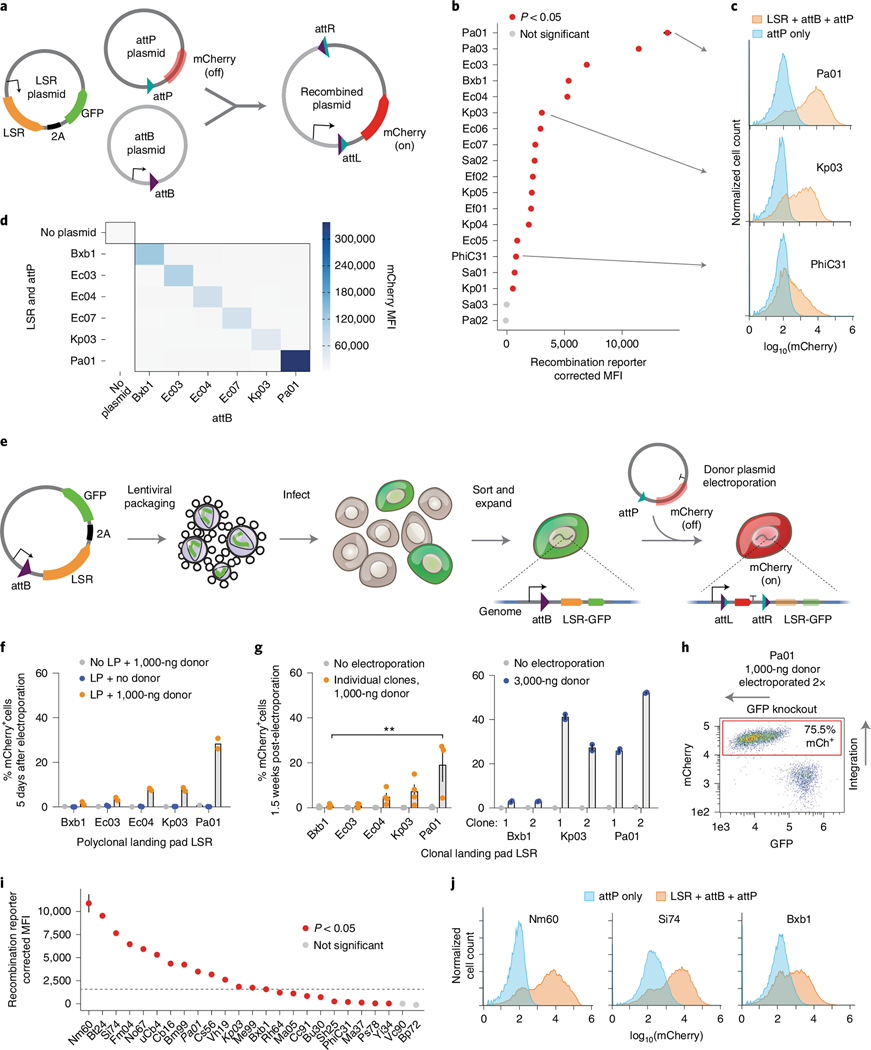
Development of efficient recombinases for landing pads. **a**, Schematic of plasmid recombination assay. Cells are co-transfected with three plasmids, and, upon recombination, mCherry gains a promoter and is expressed. **b**, Plasmid recombination assay of predicted LSRs and att sites in HEK293FT cells, shown as corrected mCherry MFI. Error = s.d. (*n* = 3). *P* value was determined by one-tailed *t*-test. **c**, Example mCherry distributions for all three plasmids (LSR + attB + attP) compared to the attP-only negative control. **d**, Plasmid recombination assay between pairs of LSR + attP and attB in K562 cells (*n* = 1). **e**, Schematic of genomic landing pad assay. An EF-1α promoter, attB and LSR are integrated via lentivirus. Upon attP donor transfection and successful integration into the landing pad, mCherry is expressed, and the LSR and GFP are displaced and knocked out. **f**, Donor integration into polyclonal genomic landing pad (LP) K562 cell lines, measured after 5 days (*n* = 2 independently transduced and then electroporated replicates). **g**, Donor integration into clonal LP cells. Asterisks show significance for comparison with Bxb1 (*P* = 0.0012, one-way ANOVA, *n* = 3 clonal cell lines for Pa01 and *n* = 4 clonal cell lines for others at 1,000-ng dose, error = s.e.m.). **h**, Pa01 clonal LP line electroporated twice in rapid succession. **i**, Plasmid recombination assay for a new batch of LSRs selected for higher quality (Methods) in HEK293FT cells, shown as corrected mCherry MFI. Error = s.d. (*n* = 3 transfections). Controls are labeled in bold, and the previous batch is in italics. The dotted line indicates the positive control Bxb1. *P* value was determined by one-tailed *t*-test. **j**, Representative mCherry distributions for all three plasmids (LSR + attB + attP) compared to the attP-only negative control.

One valuable application for LSRs is the integration of genetic cargo into a pre-installed genomic landing pad site. To explore the utility of our computationally predicted LSRs for this purpose, we selected an initial batch of candidates from predicted phage elements with evidence of independent integrations into a single site. We synthesized human codon-optimized LSR genes and their predicted attP and attB sequences and validated recombination activity in HEK293FT human cells via a plasmid recombination assay (Fig. 2a). In this assay, the attP plasmid contains a promoterless mCherry gene, which gains a promoter upon recombination with the attB plasmid, resulting in a recombination reaction product containing the sequence of both input plasmids and exhibiting mCherry fluorescence. Out of 17 candidates, we identified 15 functional LSRs (88% validation rate), defined as having greater mCherry mean fluorescence intensity (MFI) values and a greater percentage of mCherry^+^ cells than their attP-only controls (Fig. 2b,c and Supplementary Fig. 2a). Thirteen candidates had favorable recombination efficiency relative to PhiC31, whereas three were superior to Bxb1 (Fig. 2b and Supplementary Table 2). We next tested orthogonality for a subset of 5 LSRs, chosen for their diversity and favorable plasmid recombination activities, and found that they are highly orthogonal, only integrating efficiently into their cognate attachment sites and not into those of the other LSRs (Fig. 2d).

To test the efficiency of integration into the human genome, we next generated landing pad cell lines in K562 cells by using lentivirus to integrate a pEF-1α-attB-LSR-T2A-GFP cassette into the genome. We used a high dose of lentivirus, which integrates semi-randomly, such that most cells would have one or more copies of the landing pad at various genomic positions. This design enables a promoter trapping approach where successful recombination of the 3-kb attP donor plasmid would lead to gain of mCherry expression and loss of LSR and GFP expression (Fig. 2e). All five of the tested LSRs integrated with measurable efficiency, ranging from 3% to 30%, whereas Bxb1 integrated at only ~1.5% efficiency (Fig. 2f). Next, we assessed the expression stability of these polyclonal landing pad cell lines. Although GFP expression in landing pads such as Pa01 remained constant over time, others lost GFP expression, suggesting that the landing pad can sometimes be transcriptionally silenced or genetically unstable in an attB-dependent or LSR-dependent manner (Supplementary Fig. 2b). Overall, these results demonstrate that these new LSRs can efficiently integrate donor cargo into human chromosomal DNA at landing pads.

Landing pad integration in clonal lines would enable a single, consistent genomic context for donor insertion. To develop single-position landing pad lines, we integrated the landing pad LSR-GFP construct via low multiplicity of infection (MOI) lentiviral infection, expanded GFP^+^ clones after single-cell sorting and assessed clonal GFP stability, again finding that Pa01 lines are particularly stable (Supplementary Fig. 2c). We then electroporated four clones per LSR with an attP-mCherry donor plasmid. We tested four integrase candidates and found that Pa01 performed markedly better than Bxb1 across multiple independent clones in terms of the percentage of cells that were mCherry^+^ after 1.5 weeks (average of 19% versus 1%) (Fig. 2g, left). With a tripled donor DNA dose (3,000 ng), Pa01 reached 52% efficiency, whereas Bxb1 increased to only 3% integration (Fig. 2g, right). Electroporating cells with donor plasmid twice in rapid succession increased integration efficiency to over 75%, suggesting that efficiency is primarily limited by donor delivery (Fig. 2h). These cells were also GFP^−^, consistent with the desired outcome of an mCherry donor being integrated into the landing pad while simultaneously displacing and knocking out the LSR-GFP cassette. We note that each lentiviral-generated clonal line is expected to have a different landing pad genomic position, and that position could affect LSR integration efficiency. Our result that Pa01 outperforms Bxb1 in the clonal landing pad assay is consistent with the polyclonal landing pad and plasmid recombination assays, which are not vulnerable to potential position biases.

Experimental truncation of the Bxb1 attB sequence has revealed that a minimal 38-bp sequence is necessary for integration^30^, although our computational pipeline conservatively predicts 100-base pair (bp) attB sequences initially. Reasoning that shorter attachment sites would facilitate the installation of landing pads with methods such as homology-directed repair (HDR) and prime editing^31,32^, we next set out to identify the minimal attB sequences necessary for recombination. We determined a minimum 33-bp attB for efficient Pa01 recombination (Supplementary Fig. 2d) and observed efficient recombination for Kp03 down to a 26-bp attB (Supplementary Fig. 2e). At such short lengths, these attachment sites could easily be installed with cloning and cell engineering methods.

We reasoned that the orthogonality of these LSRs could be useful for multiplex gene integration (Fig. 2d). Bxb1 and other LSRs have been shown to contain a modular dinucleotide core in their attachment sites, enabling one LSR to direct the insertion of multiple cargos, each into a particular landing pad site specified by its cognate core dinucleotide^30,33,34^. We demonstrated a similar ability to substitute core dinucleotides using the plasmid recombination assay for one of the new recombinases, Kp03 (Supplementary Fig. 2f).

We then investigated the specificity of four of the new LSRs for their attachment sites by transfecting LSRs and mCherry donors into unmodified K562 cells that do not contain landing pads. We measured mCherry expression over time, reasoning that episomal donor plasmid will be diluted over time, whereas the genomic integration signal is expected to remain stable. At day 18, Pa01 showed no evidence of integration above background, indicating a lack of off-target activity, whereas Kp03 did have elevated percentages of mCherry^+^ cells, suggesting that it has off-target pseudosites (Supplementary Fig. 2g). To identify these sites, we modified a one-sided polymerase chain reaction (PCR)-based next-generation sequencing (NGS) assay for use as an LSR integration site mapping assay by priming on the inserted donor to identify genomic sequences on the other side of the donor–genome junction (Supplementary Fig. 2h,i)^35,36^. First, we quantified the percentage of off-target integrations relative to on-target integrations in landing pad cell lines (Supplementary Fig. 2j and Supplementary Table 3). This assay detected off-target integrations for all LSRs, including Bxb1 (1.34%), Pa01 (1.35%) and Kp03 (36.3%). Additionally, in wild-type cells, we enriched for genomic integrations with puromycin selection to develop target site sequence motifs from precise integration sites that were reproducible across biological replicates (Supplementary Fig. 2k and Supplementary Table 3). These motifs validated the experimentally determined minimal attachment site length and demonstrated the highly conserved dinucleotide core. Together, these results establish Pa01 as a more efficient specific landing pad LSR in comparison to Bxb1 and define the specificity and off-target profiles of additional landing pad LSRs.

Finally, we selected a second batch of 21 LSRs from our database, prioritizing those with low BLAST similarity between their predicted attB/P sites and the human genome, and applying stringent quality thresholds (Methods). We found that 17 of 21 recombinases (81%) were functional in the plasmid recombination assay, providing further validation of the computational pipeline for identifying functional candidates. Promisingly, 16 candidates were more efficient than PhiC31, whereas 11 were superior to Bxb1 (Fig. 2i,j). Our fluorescence integration assay and integration site mapping in wild-type cells identified several further LSRs with minimal off-target integrations, nominating Si74 and No67 as two top LSRs with high recombination efficiency and genomic specificity (Supplementary Fig. 2l).

### Landing pad LSRs enable parallel reporter assays

**Fig. 3 |:**
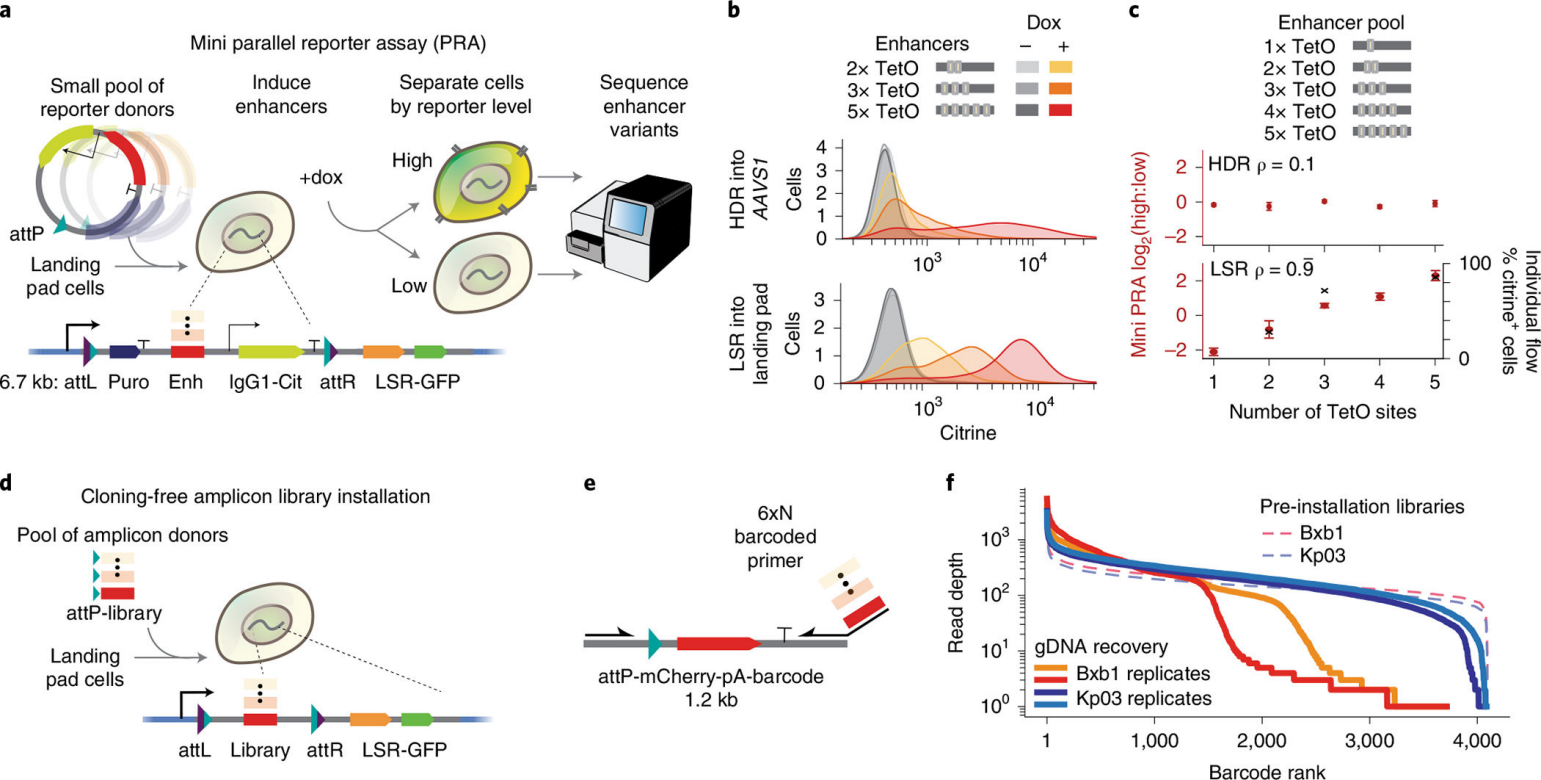
PRAs and amplicon library installation with landing pad recombinases. **a**, Schematic of mini PRA. A pool of reporter plasmids with varied synthetic enhancers containing TetO sites is integrated into the landing pad by the LSR, selected for using puromycin, and then reporter activation is induced using doxycycline, which causes rTetR-VP48 to bind TetO. Highly activated and lowly activated cells are magnetically separated; the enhancers are sequenced from gDNA in each cell population; and a ratio of reads is computed as a measurement of enhancer strength. **b**, Individual enhancer reporters with a varied number of TetO transcription factor binding sites were integrated into the AAVS1 safe harbor by HDR or into the landing pad using the Kp03 LSR. Flow cytometry measurements were taken 2 days after induction with doxycycline. Due to varied voltage settings on the cytometer, the *x*-axes are not comparable in absolute terms (*n* = 1 cell line replicate, and a second replicate is shown in Supplementary Fig. 3a). **c**, A small pooled library of synthetic enhancer reporters was integrated into the AAVS1 safe harbor by HDR or a clonal landing pad by the Kp03 LSR and measured by separation and sequencing (*n* = 2 integration replicates for HDR; *n* = 3 integration replicates for LSR; dots show the mean; error = s.d.). ρ is the Spearman correlation between the PRA measurement of enhancer strength and the number of TetO sites in the enhancer. For the LSR, pooled measurements (left *y*-axis, red circles) correlate with the percentage of citrine^+^ cells from individual reporter assays (right *y*-axis, black x, Pearson’s *r* = 0.94). **d**, Schematic for a cloning-free strategy to install libraries. A linear dsDNA library of elements containing the attP site is generated by PCR and directly delivered to landing pad cells. **e**, Schematic of an amplicon library generated by PCR from an attP-mCherry-pA template, where the reverse primer contains a 6×N barcode. **f**, Distribution of barcodes in the initial amplicon libraries (read depths 216–272×) and in gDNA extracted from cells 7 days after electroporation with 750 ng of amplicon (read depths 290–357×). dox, doxycycline.

Parallel reporter assays (PRAs) have recently become an effective means of studying libraries of diverse molecular elements, including enhancers, promoters and untranslated regions^37–39^. However, PRAs can be adversely affected by genomic position effects or other forms of heterogeneity in delivery^40^, so methods for efficient and stable integration of PRA reporters into a single genomic position are needed^41–43^. Current LSR landing pad systems are limited by recombinase efficiency, with Bxb1 integrating libraries at rates between 5% and 10%^43,44^. We reasoned that more efficient recombinases could enable larger pooled screens with better coverage and lower noise using the same number of cells. To explore the utility of the new landing pad LSRs in functional genomics, we established a mini PRA that tests the capacity of synthetic enhancers to activate a transcriptional reporter integrated in the genome (Fig. 3a).

First, we individually integrated reporters containing a varied number of rTetR-VP48 transcription factor binding sites into a Kp03 landing pad clonal line in K562 cells. This strategy of low MOI lentiviral delivery and clonal selection has previously been shown to be effective for generating single-copy Bxb1 landing pad lines for PRAs^43^. As a control, we also used HDR to integrate matched reporters into the AAVS1 safe harbor in cells lacking the landing pad. We chose this locus because HDR has variable efficiency across loci and effective TALEN reagents, and HDR protocols were previously established for this locus^45^. Our reporter gene has two components: a fluorescent citrine and an IgG1 Fc synthetic surface marker (Fig. 3a). The surface protein enables scalable and rapid magnetic bead separation of highly expressing and lowly expressing cells; for example, one can separate ~1 × 10^9^ cells in ~90 minutes (compared to ≥20 hours by fluorescence-activated cell sorting (FACS))^46^. As expected, we found that both HDR-integrated and LSR-integrated reporters were activated to degrees corresponding with the number of transcription factor binding sites in the enhancers (Fig. 3b).

Next, to test reporters in a parallelized fashion, we integrated a pooled library of reporters and performed a PRA by quantifying the abundance of each enhancer in high versus low reporter-expressing cells using NGS (Fig. 3a,c and Supplementary Fig. 3a–c). For the HDR-installed libraries, we did not see the expected positive correlation between enhancer activation strength and number of transcription factor binding sites (ρ = 0.1), which could be due to integration of multiple library members at more than one AAVS1 allele per cell (Fig. 3c)^47^. Meanwhile, for the LSR-installed libraries in clonal landing pad cells, we saw the expected correlations between the enhancer activation strength and the number of transcription factor binding sites (ρ = 0.99) and also between the PRA and individual reporter measurements by flow cytometry (*r* = 0.94; Fig. 3c). We cannot rule out the possibility that the HDR-based strategy could be optimized to yield similar results or that the results would correspond better if both methods targeted the same genomic position. Taken together, these PRA results demonstrated that landing pads can be useful for making parallelized quantitative measurements of a library of reporters and indicated that these new landing pad LSRs could enable diverse functional genomics research applications.

### Landing pad LSRs enable amplicon library installation

Pooled genetic screens currently involve large-scale plasmid library cloning followed by laborious lentiviral packaging, titering and delivery^48^. Less commonly, a recombinase landing pad can be used to integrate the cloned plasmid library into a single genomic location^49^. To overcome this cloning step, we developed a proof of concept for a new method for installing PCR amplicon libraries directly into cells using landing pad recombinases. Although LSRs are canonically thought to integrate circular donors, it was recently observed that PhiC31 can integrate linear PCR amplicon donors into genomic DNA (gDNA)^24^. We confirmed this method by transfecting a linear donor with and without PhiC31 and performing junction PCR outwards from the ends of the donor (Supplementary Fig. 3d). We found short indels in the terminal portions of the original linear DNA, suggesting that linear donor ends are likely joined by NHEJ-based DNA repair machinery in an LSR-independent manner (Supplementary Fig. 3e,f).

We sought to exploit linear donor integration for a rapid library installation method, directly transfecting a PCR amplicon library into Kp03 and Bxb1 landing pad cells (Fig. 3d). Here, we used an increased library size compared to the previous PRA (Fig. 3d). First, we generated an amplicon consisting of a promoterless attD-mCherry followed by a library of 4,096 barcodes surrounded by flanking sequences (Fig. 3e). We observed that the new recombinase Kp03 was ~10× more efficient than Bxb1 at integrating this linear donor (Supplementary Fig. 3g), with an efficiency of ~3% (Supplementary Fig. 3g,h). Barcode sequencing from gDNA cross-junction PCR revealed that the improved efficiency of the new LSR Kp03 directly translated into more uniform recovery of library barcodes (Fig. 3f and Supplementary Fig. 3i–l). The increase in integration efficiency for Kp03 over Bxb1 overcame a major limitation by reducing barcode dropout from 22% with Bxb1 to only 1% with Kp03.

### Human genome-targeting LSRs can integrate at predicted sites

Up until this point, we relied on pre-engineering cell lines with landing pads, which requires two DNA delivery steps. However, our work in defining attachment sites and pseudosites in the human genome raised the possibility that we could use these systems to integrate payloads directly into the human genome at one or more safe locations without pre-engineering. Direct genome integration could be very useful for applications such as in vivo gene therapy. Historically, the integration sites of LSRs such as PhiC31 had to be experimentally characterized in human cells. Given the expanded size of our LSR database with defined attB and attP sequences, we reasoned that we could first computationally search for LSRs that naturally target an attachment site highly similar to a sequence in the human genome.

**Fig. 4 |:**
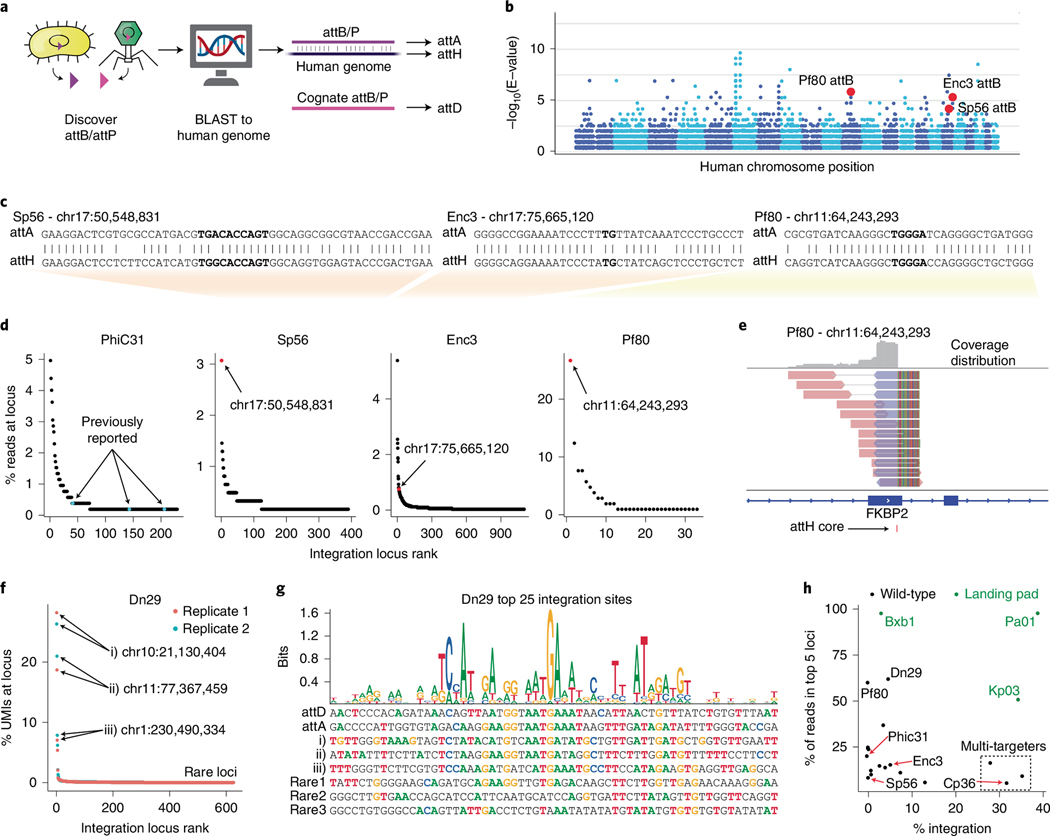
Discovery of specific human genome-targeting recombinases. **a**, Predicted attB and attP sequences were searched against the human genome using BLAST. The attachment site with the best match to the human genome is denoted attA (acceptor), and the corresponding human target site is denoted attH (human). The cognate attachment site is denoted attD (donor). **b**, BLAST hits of attB and attP sites that are homologous to sequences in the human genome. All hits that meet E < 1 × 10^−3^ are shown. The 22 autosomal chromosomes are shown in numerical order from left to right in alternating colors. **c**, Alignments of the microbial attachment sites (attA) to the predicted human attachment sites (attH) for three candidates. The attachment site center is bolded, representing the portion of the native attP and attB that is identical. **d**, Detected integration loci, ranked according to the number of uniquely mapped reads. Blue points are previously reported integration sites for PhiC31, and red points indicate predicted integration sites for Sp56, Enc3 and Pf80. **e**, Reads at the top integration site. Reads that align in the forward direction are shown in red, and those aligning in the reverse direction are shown in blue, with a gray line connecting paired reads. **f**, Detected integration loci for Dn29. UMIs were incorporated into the donor plasmid. The top three integration sites and sites with only one detected UMI (‘rare’) are highlighted. Results of three biological replicates are shown. **g**, A target site motif for Dn29 calculated using the top 25 target sites in K562 cells. Example integration sites are shown below, including the top three integration sites and three sites with only one detected UMI (rare1, rare2 and rare3). Colored nucleotides match the most common nucleotide at that position in the top 25 sites. **h**, LSR integration specificity and efficiency. For wildtype cells (black), efficiency is a corrected percentage of mCherry^+^ cells 18 days after electroporation with an LSR and donor plasmid. For landing pad cells (green), efficiency is the mean of mCherry^+^ cells in all clones (from Fig. 2g, right). To estimate specificity, UMI counts were used if available; otherwise, uniquely mapped read counts were used, and counts were merged across replicates.

We used BLAST to search all attB/P sequences against the GRCh38 human genome assembly (Fig. 4a) and identified 856 LSRs with a highly significant match for at least one site in the human genome (BLAST E-value <1 × 10^−3^; Fig. 4b,c). We synthesized 101 LSRs prioritized by high BLAST match quality in the plasmid recombination assay and confirmed that 25 candidates recombined at the predicted attachment sites (one-tailed *t*-test, *P* < 0.05; Supplementary Fig. 4a). We found that 21 of 37 (56.75%) high-quality candidates recombined as predicted, in contrast to four of 64 (6.25%) low-quality candidates. We named the attP and attB sites according to their BLAST hits, with the attachment site that BLAST aligned to the human genome being renamed to attA (acceptor), and the other being renamed to attD (donor). The predicted target site in the human genome was renamed to attH (human) (Fig. 4a,c), and we confirmed that several of our candidates recombined with their predicted attH sequence in the plasmid recombination assay by replacing attA with attH on the acceptor plasmid (Supplementary Fig. 4b).

Next, we mapped their integration sites in the human genome to test our computational predictions, using the same NGS-based integration site mapping assay as above. As a control, we mapped integration sites for PhiC31 and found integration into three previously reported integration sites^50^ as well as 216 additional sites (Fig. 4d and Supplementary Table 4). As for the new LSRs Sp56 and Pf80, the predicted target loci by BLAST were indeed the top integration sites with the most uniquely mapped reads (Fig. 4d,e and Supplementary Fig. 4c). For Enc3, the predicted target site was among the top 20 integration loci, although it was not the most frequently targeted locus (Fig. 4d and Supplementary Fig. 4d). Of the tested LSRs, Pf80 had the highest specificity, with 34.3% of unique reads mapping to the predicted target site (Fig. 4d,e).

An ideal genome-targeting LSR would integrate with robust efficiency in a site-specific manner. The genome-targeting candidates that we tested exhibited varying levels of efficiency, with Enc3 and Dn29 in particular having considerably higher efficiency (6% and 5%, respectively) than PhiC31 or Pf80 (both <1%; Supplementary Fig. 4e). For Dn29, we found that 61.9% of integrations occurred in just the top five target sites (Fig. 4f–h and Supplementary Table 4), which were found in intronic or intergenic regions (Supplementary Fig. 4f). We identified a Dn29 target site motif that was much more similar to its original attB and the top three integration sites than low-frequency integration sites (Fig. 4g, Supplementary Fig. 4g and Supplementary Table 4). This combination of efficiency and specificity makes Dn29 a genome-targeting candidate well-suited for further engineering and optimization (Fig. 4h). Taken together, our pipeline is able to nominate serine integrases that are likely to target the human genome, predict their target site preference and identify LSRs with superior efficiency and specificity to PhiC31.

### Multi-targeting LSRs can be efficient and unidirectional

**Fig. 5 |:**
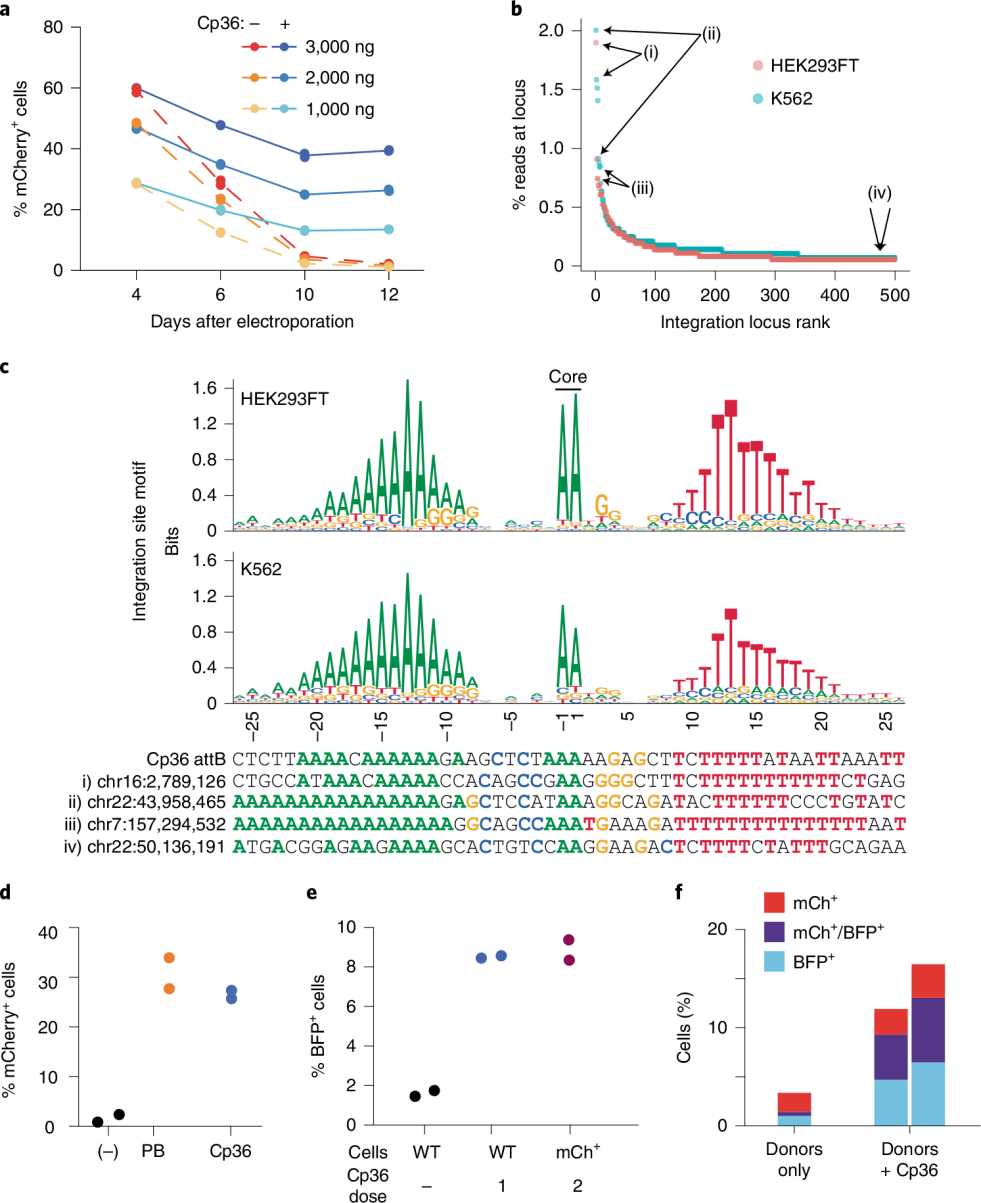
Development of efficient multi-targeting recombinases. **a**, Efficiency of the multi-targeting integrase Cp36 in K562 cells. Bxb1 paired with a Cp36 attD donor was used as a negative control. Fluorescence was measured out to 12 days after electroporation by flow cytometry. Lines show the mean (*n* = 2), with dashed lines used for negative controls. **b**, Integration site mapping assay results for Cp36. The top 500 loci across two experiments, one performed in HEK293FT cells and another performed in K562 cells, are shown. Roman numerals highlight the 3 top sites (i–iii) and one rare site (iv), and correspond with the bottom rows in **c**. **c**, Cp36 target site motifs and example target sequences. Precise integration sites and orientations were inferred at all loci, and nucleotide composition was calculated for the top 200 sites. Example integration sites specified in **b** are shown below the motifs, where nucleotides are highlighted with their respective colors if they match the consensus nucleotide. **d**, Efficiency of Cp36 and Super PiggyBac for stable delivery of mCherry donor plasmid in K562 cells. The 7.2-kb donor plasmid contains the Cp36 attD and the PiggyBac ITRs. Ec03 LSR was used as a negative control that lacks an attachment site on this donor plasmid (*n* = 2). **e**, Wild-type K562 or Cp36-dosed mCherry^+^ and puromycin-selected cells were transfected with 2,000 ng of a second fluorescent reporter (mTagBFP2) and analyzed by flow cytometry 13 days after electroporation (*n* = 2). Dash shows negative control treated with BFP donor only. **f**, Flow cytometry 12 days after electroporation of both fluorescent donors and Cp36 plasmids into K562 cells. Negative control cells were transfected with the donors and pUC19 (*n* = 2 replicates shown as stacked bars for Cp36 condition).

Some serine recombinases have evolved transposition or multi-targeting capabilities, allowing them to target many different attB sites in a given prokaryotic genome^51^. Efficient insertion into a defined series of pseudosites would be very useful for transgene integration relative to semi-random integrases, such as lentivirus, Piggybac or Sleeping Beauty transposase. To explore this, we tested an LSR from *Clostridium perfringens*, found in the multi-targeting clade (Fig. 1d,g) in our database, which we named Cp36. Cp36 successfully integrated an mCherry donor cargo into the genome of K562 and HEK293FT cells at up to 40% efficiency without pre-installation of a landing pad or antibiotic selection (Fig. 5a and Supplementary Fig. 5a).

Using the integration site mapping assay, we identified over 2,000 unique integration sites for Cp36 with the top ten loci accounting for 8.27% and 11.4% of uniquely mapped reads in HEK293FT cells and K562 cells, respectively. (Fig. 5b, Supplementary Fig. 5b and Supplementary Table 5). Across these two cell types, we report high correlation between the top integration sites (Pearson’s *r* = 0.45, *P* = 0.0002; Supplementary Fig. 5c). We observed modest integration site enrichment in DNase hypersensitivity peaks for Cp36 and three other multi-targeters that we tested, suggesting that chromatin accessibility can influence integration efficiency, but the effect size is generally small (Supplementary Fig. 5d). Next, we constructed a sequence motif for Cp36 target sites, which is composed of an A-rich 5′ region, an AA dinucleotide core and a 3′ T-rich region (Fig. 5c and Supplementary Table 5). This motif built from Cp36 integration sites corresponds well with the motif prediction built from LSRs in the same 50% amino acid identity cluster as Cp36 and their cognate attB sites in our database (Supplementary Fig. 5e and Supplementary Table 5). Upon further analysis, we found that sequence motifs built from database-derived attB sequences often perform well at predicting experimentally observed integration sites (area under the receiver operating characteristic curve (AUROC) = 0.94 for Cp36 and AUROC = 0.44–0.84 for *n* = 7 other LSRs; Supplementary Note 2 and Supplementary Fig. 6).

We next compared Cp36 to the Super PiggyBac transposase, a common tool for delivering DNA cargoes semi-randomly into TTAA tetranucleotides found in a target genome. We designed a 7.2-kb plasmid construct that included a Cp36 attD site, PiggyBac inverted terminal repeat (ITR) sequences and an mCherry reporter to directly compare the efficiencies of these two enzymes (Supplementary Fig. 5f). We found that Cp36 performs at similar efficiencies to PiggyBac (26.6% and 30.9% of cells with stable integration, respectively) (Fig. 5d), despite Cp36 comprising an unaltered microbial protein sequence and Super PiggyBac being an engineered, hyperactive version of the transposase intended for genome engineering^52^.

PiggyBac is a bidirectional integrase and excisionase, resulting in both excision and local hopping of cargo upon repeated treatment of cells^53^. Excision activity is undesirable for serial delivery applications, so we sought to assess the directionality of Cp36 recombinase. First, we re-dosed Cp36 into mCherry^+^ cells generated using Cp36 and an mCherry donor. Stable mCherry signal implied a lack of excisionase activity, a finding that aligns with numerous reports on LSR unidirectionality (Supplementary Fig. 5g)^54^. To test if Cp36 could be re-used to integrate a second cargo, we generated a population of mCherry^+^ cells via Cp36-mediated integration and puromycin selection and re-electroporated with Cp36 and a donor encoding BFP. After 13 days, we found that 9% of the cells were double positive (mCherry^+^ and BFP^+^) (Fig. 5e and Supplementary Fig. 5h), with 100% of cells retaining mCherry expression (Supplementary Fig. 5i), demonstrating that a second gene could be delivered without losing the first cargo. To confirm that Cp36 is unidirectional, we replaced the attB and attP sequences with attL and attR in the plasmid recombination assay, observing no recombination between attL and attR (Supplementary Fig. 5j,k). Furthermore, we found that simultaneous delivery of Cp36 with both mCherry and BFP fluorescent reporter donors resulted in stable populations expressing both markers (Fig. 5f), suggesting that Cp36 could be used to generate cells with multi-part genetic circuits in a single transfection.

Finally, we demonstrated that two other multi-targeting LSRs from our pipeline, Pc01 and Enc9, integrated cargoes into the human genome with efficiencies of 13.3% and 35.5%, respectively (Supplementary Fig. 5l), demonstrating that this multi-targeting clade is a rich repository of efficient recombinases. These results reveal the existence of a subset of LSRs, not previously tested in eukaryotic cells, with highly efficient, unidirectional integration activity and longer targeted DNA motifs (≥20 bp) compared to lentivirus or transposase systems (2–4 bp).

## Discussion

DNA-targeting enzymes derived from diverse bacterial genomes have revolutionized molecular biology and genome engineering. Due to their ability to integrate large DNA cargoes, integrase systems such as recombinases and transposases have been commonly employed for workflows such as Gateway cloning or generation of stable cell lines^55,56^. Despite longstanding efforts to adapt them for genome editing, the low efficiency and small number of known LSRs have greatly limited their broader utility for mammalian genome engineering^57^. We sought to address these challenges by systematically identifying and characterizing a large number of LSR enzymes from microbial MGEs. By increasing the number of known LSR and cognate attachment site combinations by >100-fold relative to previous work^28^, we identified three functional classes of LSRs—landing pad LSRs, human genome-targeting LSRs and multi-targeting LSRs—all of which can be used to target the human genome with potential clinical and research utility (Supplementary Table 1).

We first identified an array of new landing pad LSRs, including Kp03 and Pa01, which outperform the previously characterized Bxb1 by 2–7-fold in episomal and chromosomal integration efficiency, enabling single payload insertions at efficiencies of 40–75% without selection. These landing pad LSRs direct DNA cargoes into specific landing pad sites in an orthogonal manner dictated by the core dinucleotide in the attachment sites, meaning that multiple payloads could be specifically addressed to an array of landing pads in the same cell^30^.

We further demonstrated a new method to directly integrate an amplicon library into a landing pad in human cells. With this method, a single library element at a single site could be assessed per cell if three criteria are met: (1) the landing pad is carefully installed into a single site at a single copy (for example, using existing protocols demonstrated for Bxb1 (refs. ^43,58^)); (2) a promoter trap drives expression of only on-target landing pad-integrated library elements; and (3) a cross donor–genome junction PCR only amplifies on-target landing pad-integrated library elements for sequencing. Without these features, noise could be added to a screen. Although this method requires additional development, we think that there is potential for new higher-efficiency landing pad recombinases to accelerate functional genomics applications by obviating the need for laborious library cloning and lentiviral delivery.

We also discovered LSR variants that catalyze efficient integration directly in the human genome at a small number of well-defined endogenous genomic locations. Despite extensive characterization of PhiC31 with the goal of therapeutic gene integration^59^, its reported integration rate is under 3% across at least 42 pseudosites^50,60,61^. In our hands, PhiC31 efficiency did not surpass the ~1% background efficiency of plasmid donor integration. Dn29 integrated in 5% of cells, and 61.9% of integrations were in just the top five integration sites. Excitingly, we demonstrated the ability to predict integration sites within the human genome and identified LSRs that integrate into their top site at frequencies ranging from 6% (Sp56 and Enc3) to 34.3% (Pf80) of all integrations. This is much more specific than other microbial integrases often used in human cells, such as PiggyBac transposase^62^ or Sleeping Beauty transposase^63,64^. Our LSR database could also include candidates that could directly target non-human genomes, including plants, microbes and model or non-model organisms, potentially facilitating stable transgenesis into diverse organisms^65^.

Because our computational approach identifies candidate LSRs as well as their target sites, this expansive database provides insight into the innate targeting specificity of each LSR. Some appear to target unique sites in bacteria, whereas others are more promiscuous, a group that we describe as multi-targeting. When we introduced the multi-targeting LSR Cp36 into human cells, we found that it integrated cargo DNA into multiple sites in the human genome with high combined efficiency (>40%) at multiple sites. Cp36 compares favorably with the Super PiggyBac transposase in unidirectionality (it does not excise its previous insertions when re-used) and design (it only requires appending a short attP (50–100 bp) to one end of its cargo rather than 200–300-bp flanking arms). Super PiggyBac has been extensively used for transgenesis, mutagenesis and therapeutic purposes, such as chimeric antigen receptor (CAR) T cell engineering^66–68^. We envision that multi-targeting LSRs could supplant transposases and retroviruses in applications that require high-efficiency integration with better-defined target sites, such as cell therapies.

Interestingly, although LSRs alone perform a unidirectional integration reaction, they can perform the reverse excision reaction when co-expressed with a reverse directionality factor (RDF) protein^69,70^. An exciting future direction is to extend the bioinformatic search of these MGEs to retrieve the RDF corresponding to each of these LSRs. Such RDFs could expand LSR utility for synthetic biology and potentially provide a form of antidote or safety switch in cases where an LSR-mediated integration needs to be removed.

A potential limitation of LSRs is that they are not readily reprogrammed to target new sequences. However, there is a great diversity of LSRs across bacterial systems, as demonstrated by our database comprising over 1,000 different clusters. We found that natural attachment sequences vary widely across LSR clusters, suggesting that an LSR could likely be found to target many sequences of interest. Further work to dissect LSR structure–function relationships with their target DNA sequence could enable the design of synthetic LSRs that can be reprogrammed to target new locations in genomes, providing a simple single-effector-protein tool to integrate large cargoes into arbitrary locations. In addition, LSRs or effector domains of LSRs could potentially be combined with an RNA-directed programmable CRISPR targeting system to direct the LSR functionality toward a sequence-specific, easily programmable site. Recently, such an approach was described with prime editors^71,72^, which could be combined with the efficient and specific landing pad LSRs described here to more efficiently integrate large cargos into programmed locations.

Overall, we envision diverse applications of integrase systems for reliable, stable and unidirectional targeting of the genome, such as functional genomics screens where controlled insertion of unique library elements into unique single cells is desired^43^. Second, these landing pads could be useful in the development of engineered cells or cellular therapies, where custom combinations of genes can be introduced to induce cell-type-specific differentiation or to control cell behavior via synthetic gene circuits. Third, both endogenous and engineered LSR attachment sites can be used to record and reconstruct cell lineages during cell fate specification in development or disease models^34^. Finally, LSRs could also enable larger-scale genome engineering, including controlled models of large structural rearrangements, by installing attachment sites at distal sites in a genome^73^. Beyond LSRs, there are many more DNA mobilization genes lying in wait within massive sequence databases, providing an expansive opportunity to derive insights into their mechanisms of protein–DNA interaction and enrich the genome engineering toolbox.

### Online content

Any methods, additional references, Nature Research reporting summaries, source data, extended data, supplementary information, acknowledgements, peer review information; details of author contributions and competing interests; and statements of data and code availability are available at https://doi.org/10.1038/s41587-022-01494-w.

## Methods

### Cell lines and cell culture

Experiments were carried out in K562 cells (American Type Culture Collection, CCL-243) and HEK293FT cells. K562 cells were cultured in a controlled humidified incubator at 37 °C and 5% CO_2_ in RPMI 1640 (Gibco) media supplemented with 10% FBS (HyClone), penicillin (10,000 IU ml^−1^), streptomycin (10,000 μg ml^−1^) and L-glutamine (2 mM). HEK293FT cells, as well as HEK293T and HEK293T-LentiX cells used to produce lentivirus, as described below, were grown in DMEM (Gibco) media supplemented with 10% FBS (HyClone), penicillin (10,000 IU ml^−1^) and streptomycin (10,000 μg ml^−1^).

### Computational workflow to identify thousands of LSRs and cognate attachment sites

The LSR identification workflow was implemented as described schematically in Fig. 1a. In total, 146,028 bacterial isolate genomes available in the National Center of Biotechnology (NCBI) RefSeq database were identified on 22 August 2019. Genomes were then clustered at the species level using the NCBI taxon ID and the TaxonKit (version 0.7.1) tool^74^. Genomes within each species were randomized and batched into sets of 50 and 20 genomes, where the first batch included 50 genomes, and all subsequent batches contained 20 genomes. Each batch was then processed by downloading all relevant genomes from NCBI, annotating coding sequences in each genome with Prodigal (version 2.6.3)^75^ and then searching for all encoded proteins that contained a predicted Recombinase Pfam domain (PF07508) using HMMER (version 3.3.2)^76,77^. Genomes that contained a predicted LSR were then compared to genomes that lacked that same LSR using the MGEfinder (version 1.0.6) command ‘wholegenome’, which was developed for this purpose by adapting the default MGEfinder tool to work with draft genomes^27^. If MGE boundaries that contained the LSR were identified, all of the relevant sequence data were saved and stored in a database. The workflow was parallelized using Google Cloud virtual machines.

After this initial round of LSR discovery was complete, a modified approach was taken to further expand the database and avoid redundant searches. First, bacterial species with a high number of isolate genomes available in the first round were analyzed to determine if further inspection of these genomes would be necessary. Rarefaction curves representing the number of new LSR families identified with each additional genome analyzed were estimated for these common species, and species that appeared saturated (that is, fewer than one new LSR cluster per 1,000 genomes analyzed) were considered ‘complete’, meaning no further genomes belonging to this species would be analyzed. Next, 48,557 genomes that met these filtering criteria were downloaded from the GenBank database and prepared for further analysis. The analysis was very similar to round 1 but with some notable differences. First, a database of over 496,133 isolate genomes from the RefSeq and GenBank genomes was constructed. PhyloPhlAn (version 3.0.2) marker genes were then extracted from all of these genomes^78^. Next, for each genome that was found to contain a given LSR, closely related isolates found in the database were selected according to marker gene homology and used for the comparative genomics analysis and further LSR discovery. This marker gene search approach was made available in a public GitHub repository (https://github.com/bhattlab/GenomeSearch). This second round of LSR and attachment site discovery increased the total number of candidates by approximately 32%.

For each identified LSR, two attB sequences could be chosen to represent the original attB sequence, either through concatenation of the sequences immediately flanking the MGE on the post-integration chromosome or through using the sequence as it exists on the pre-integration chromosome—sequences that could differ from each other slightly. In this study, the sequences flanking the element on the post-integration chromosome were used, motivated by the reasoning that this sequence would more closely represent the original attB as it existed immediately before integration. A sequence spanning 50 bp around the attachment site ‘center’, defined as the short stretch of sequence that was homologous between attB and attP, was used to represent both attB and attP sequences.

### Predicting LSR target site specificity

LSR sequences were clustered at 90% or 50% identity using MMseqs2 (version 13–45111)^79^. Protein sequences that overlapped with predicted attachment sites were extracted from their genome of origin and clustered with all other target proteins at 50% identity using MMseqs2. LSR attachment site combinations that were found to meet quality control filters were considered. To identify site-specific LSRs, only LSRs clustered at 50% identity and target genes clustered at 50% amino acid identity were considered. Next, LSR target pairs were filtered to only include target gene clusters that were targeted by three or more LSR clusters. Next, only LSR clusters that targeted a single target gene cluster were considered. The remaining sets of LSR clusters were considered to be single-targeting, meaning that they were thought to site-specifically target only one gene cluster. Multi-targeting or transposable LSRs with minimal site specificity were identified. Only LSRs clustered at 90% identity and target genes clustered at 50% amino acid identity were considered. Next, the total number of target gene clusters that were targeted by each LSR cluster was counted, and LSR clusters that targeted only one gene cluster were removed from consideration. Next, the remaining LSRs were binned according to the number of protein clusters that they targeted. For the purposes of this paper, ‘>3’ target gene clusters is considered fully multi-targeting. Each 50% identity LSR cluster was then assigned to a multi-targeting bin according to the highest bin attained by any one 90% LSR cluster found within the 50% identity LSR cluster.

### Phylogenetic tree construction

Representative sequences of each quality-controlled 50% identity LSR cluster were used to construct the phylogenetic tree. LSRs were aligned using MAFFT in G-INS-i mode (version 7.471)^80^, and IQ-TREE (version 2.1.2) was then used to generate a consensus tree using 1,000 bootstrap replicates and automatic model selection^81^.

### Phylogenetic analysis of site-specific integrases targeting a conserved attachment site

One example of several site-specific integrases targeting a conserved attachment site is shown in Fig. 1e. All attB attachment sites were clustered at 80% identity using MMseqs2 (ref. ^82^). Candidates were filtered to include only those that met quality control thresholds and then attB sites that were ranked by the number of LSR clusters that were found to target them. An example attB cluster was chosen for further analysis. All LSRs that targeted this attB cluster were extracted from the database and were aligned using the MAFFT L-INS-i algorithm^80^. Amino acid identity distances between all LSRs were calculated, and the distance matrix was used to create a hierarchical tree in R. LSRs that were 99% identical at the amino acid level or more were collapsed into a single cluster. This hierarchical tree was visualized and shown in Fig. 1e, along with all attB sites that were targeted by the LSRs.

### Identifying target site motifs from attachment sites in the LSR database

The attachment sites associated with multi-targeting LSRs in the database were analyzed to determine target site motifs, as shown in Fig. 1h and Supplementary Fig. 1e. Multi-targeting LSRs in the database were analyzed at the level of individual orthologs, at the level of 90% amino acid identity clusters and at the level of 50% amino acid identity clusters. For each of these levels, only candidates that were found to target more than ten unique attB sequences or ten target genes clustered at 50% amino acid identity were kept. Then, all of the corresponding attB sequences were extracted, with only one attachment site per target gene cluster being extracted to avoid redundancy. These attB sequences were then initially aligned using MAFFT L-INS-i (ref. ^80^). Next, possible core dinucleotides were identified in each alignment by extracting all dinucleotides in the alignment and ranking them by the conservation of their most frequent nucleotides and their proximity to the center of the attB sequences, using a custom score that equally weighted high nucleotide conservation and normalized distance to the attB center. Candidates were then re-aligned only with respect to these predicted dinucleotide cores rather than using an alignment algorithm, such as MAFFT. These alignments were then visualized in R using ggseqlogo (version 0.1) to identify conserved target site motifs^83^.

### Annotation of LSR-carrying MGEs

Several tools and approaches were used to annotate LSR-carrying MGEs. Phages/prophages were identified using VirSorter2 (version 2.2.3), keeping predictions with boundaries that covered at least 75% of the MGE (ref. ^84^). ICEs and IMEs were identified using several approaches. First, conjugative elements were identified using CONJscan (version 1.0.2)^85^ profile HMMs and hmmsearch^77^, annotating an element as a conjugative element if it contained at least one VirB, T4CP and MOB protein using an E-value cutoff of 1 × 10^−4^. Next, MGEs were aligned to ICEberg (version 2.0)^86^ elements using blastn (version 2.12.0)^87^, identifying elements as ICE/IME elements if they shared at least 80% nucleotide identity and at least 75% alignment coverage with an ICEberg element. Plasmids and other replicons were identified by aligning elements to PLSDB (ref. ^88^) plasmids using blastn, identifying elements as plasmids if they shared at least 80% nucleotide identity and at least 75% alignment coverage with one of these plasmids. Other replicons were identified if they encoded proteins that matched (E-value = 1 × 10^−4^) any of the following Pfam^89^ profile HMMs: Bac_RepA_C, IncFII_repA, RepA_C, RepA_N, RepL, Rep_1, Rep_2, Rep_3, Rep_trans, Rol_Rep_N and TrfA. For annotating the MGE of origin for the LSR clusters presented in Supplementary Fig. 1a, we assigned MGE categories using plurality voting of all relevant MGE annotations, with ties being resolved in the following order: dsDNA Phage, ICE/IME, Plasmid and Other replicon. MGEs with no annotations were assigned to the ‘Other’ category.

### Target gene Pfam enrichment, Gene Ontology term enrichment and anti-phage analysis

Target genes, or genes that were found to be targeted and disrupted by LSRs upon integration, were annotated using the Pfam-A profile HMM models^89^. One representative sequence per target gene cluster, clustered at 50% identity using MMseqs2 (ref. ^79^), was selected and analyzed. Only target genes that were targeted by LSRs outside of the multi-targeting clade (Fig. 1b) were considered. Randomly selected background genes were chosen from the contigs on which each target gene was found, and these background genes were also analyzed using Pfam domain models. A two-sided Fisher’s exact test was then used to identify Pfam domains that were enriched among target genes over background genes, only calculating enrichment for Pfam domains that occurred in at least five different target genes. The false discovery rate (FDR)-adjusted *P* values were calculated by running the p.adjust R command on all the Fisher’s exact test *P* values.

For Gene Ontology (GO) term enrichment, InterProScan version 1.8.0_152-release was used to map target genes and background genes to relevant GO terms^90^. Enrichment of specific GO term pathways was calculated using the two-sided Fisher’s exact test as was done for individual Pfam domains, testing only terms that mapped to at least five different target genes.

Anti-phage defense gene enrichment was determined using a different approach. First, genomes that contained target genes were annotated using DefenseFinder (version 1.0.8)^91,92^. These annotations were used to identify any target genes that were also predicted to be anti-phage defense genes. Next, reasoning that if target genes were enriched within or near anti-phage systems then that would indicate an evolved LSR integration strategy, we calculated the distance between target genes and the nearest anti-phage defense gene. This distribution of distances was then compared with the distances between randomly selected genes and the nearest defense gene. The difference between these two distributions was calculated using a Wilcoxon rank-sum test in R.

### Initial landing pad LSR candidate selection

LSRs for the initial set of 17 landing pad candidates were identified by searching for the Recombinase Pfam domain (PF07508) among the MGEs that we previously identified^27,76^. The identity of the attachment site was inferred from the boundaries of the MGE that contained each LSR. For example, imagine a sequence of nucleotides that has the following structure:

$${\text{B}}_{1}-\text{D}-{\text{P}}_{1}-\text{E}-{\text{P}}_{2}-\text{D}-{\text{B}}_{2}$$

where B_1_ indicates the sequence flanking the MGE insertion on the 5′ end; D indicates the target site duplication created upon insertion (if it exists); P_1_ indicates the terminal sequence flanking the 5′ integration boundary that is included in the MGE; E is the intervening MGE; P_2_ indicates the terminal sequence flanking the 3′ integration boundary that is included in the MGE; and B_2_ indicates the sequence flanking the MGE insertion on the 3′ end. Then, the attB and attP sequences can be reconstructed as:

$$\mathrm{attB}=B_{1}+D+B_{2}$$

$$\text{attP}=P_{2}+D+P_{1}$$

where the ‘+’ operator in this case indicates nucleotide sequence concatenation.

Candidates were then annotated to determine features such as (1) whether or not the element was predicted to be a phage element^93^, (2) how many isolates contain the integrated MGE and (3) how often MGEs containing distinct LSRs will integrate at the same location in the genome. Candidates were then given higher priority if they were contained within predicted phage elements, if they appeared in multiple isolates and if the attachment sites were targeted by multiple distinct LSRs. A final list of 17 candidates, listed in Fig. 2b, was then taken forward and validated experimentally.

### Subsequent selection of LSR candidates of high quality

As subsequent batches of LSRs were synthesized and tested in our various assays, we improved our quality control criteria for selecting further candidates to synthesize and assay. In our initial batch of human genome-targeting candidates, few quality control filters were put in place. Subsequent batches were more stringently quality-controlled. We settled on one set of quality control criteria that substantially increased the experimental validation rate. First, we only considered LSR-carrying MGEs that were identified through comparing genomes that shared at least 95% average nucleotide identity (ANI) as calculated using FastANI (ref. ^94^), a commonly accepted ANI cutoff for identifying members of the same species. Next, LSRs with large attachment site centers, above 20 bp in length, were removed. The attachment site center is the portion of the attB and the attP that are identical and should contain the dinucleotide core. Next, LSRs with attachment sites with more than 5% of their nucleotides being ambiguous in the original genome assemblies were removed. Next, only LSRs between 400 amino acids and 650 amino acids in length were kept. Next, only predicted LSRs that contained at least one of the three main LSR Pfam domains were retained (Resolvase, Recombinase and Zn_ribbon_recom). Next, LSRs were removed from consideration if their sequences contained more than 5% ambiguous amino acids. Next, only LSRs that were found on integrative MGEs that were fewer than 200 kb in length were retained, where larger elements were presumed to be technical artifacts. And finally, only LSRs that were within 500 nucleotides of their predicted attachment sites were retained. Candidates that met all of these filters were considered to meet quality control thresholds.

### Plasmid recombination assay to validate LSR-attD-attA predictions

Three plasmids were designed for each LSR candidate to test recombination function on an episomal reporter. The effector plasmid contains the EF-1α promoter, followed by the recombinase coding sequence (codon optimized for human cells), a 2A self-cleaving peptide and an EGFP coding sequence. The attA plasmid contains an EF-1α promoter, followed by the attA sequence, followed by mTagBFP2 coding sequence, which should constitutively express the mTagBFP2 protein in human cells. The attD plasmid includes only the attD sequence followed by the mCherry coding sequence, which should produce no fluorescent mCherry before integration of the attA plasmid. Next, 20,000 HEK293FT cells were plated into 96-well plates and transfected 1 day later with 200 ng of effector plasmid, 70 ng of attA plasmid and 50 ng of attD plasmid using Lipofectamine 2000 (Invitrogen). Then, 2–3 days after transfection of cells with all three plasmids, cells were measured using flow cytometry on an Attune NxT Flow Cytometer (Thermo Fisher Scientific) and software (version 5.1.1). HEK293FT cells were lifted from the plate using TrypLE (Gibco) and resuspended in Stain Buffer (BD) before flow. These experiments were conducted in triplicate transfections. Cells were gated for single cells using forward and side scatter and then on cells expressing fluorescent EGFP. Next, mTagBFP2 fluorescence was measured to indicate the amount of un-recombined attD plasmids, and mCherry fluorescence was measured to indicate the amount of recombinant plasmid indicating successful LSR-mediated integration. Corrected MFI was obtained by subtracting the average MFI of all matching attD-only control replicates from the average MFI of the three-plasmid transfected cells. mCherry and EGFP gating was determined based on an empty backbone transfection.

An experiment testing recombinases with matched and unmatched attB and attP plasmids was performed similarly, in K562 cells. In total, 1.2 × 10^6^ K562 cells were electroporated in 100 μl of Amaxa solution (Lonza Nucleofector 2b, program T-016), with 300 ng of the 11.6-kb LSR plasmid, 869 ng of the 4.2-kb attB plasmid and 621 ng of the 3-kb attP plasmid. Three days after transfection, mCherry MFI of ungated cells was measured by flow cytometry on a BD Accuri C6 cytometer and accompanying software (version 227).

### Landing pad cell line production

Landing pad LSR candidates were cloned into lentiviral plasmids under the expression of the strong EF-1α promoter, with their attB site in between the promoter and start codon, and with a 2A-EGFP fluorescent marker downstream the LSR coding sequence. Lentivirus production and spinfection of K562 cells were performed as follows. In each well of a six-well tissue culture plate, 5 × 10^5^ HEK293T cells were plated in 2 ml of DMEM, grown overnight and then transfected with 0.75 μg of an equimolar mixture of the three third-generation packaging plasmids (pMD2.G, psPAX2 and pMDLg/pRRE) and 0.75 μg of LSR vectors using 10 μl of polyethylenimine (Polysciences, 23966) and 200 μl of cold serum-free DMEM. pMD2.G (Addgene plasmid 12259; http://n2t.net/addgene:12259; RRID: Addgene_12259), psPAX2 (Addgene plasmid 12260; http://n2t.net/addgene:12260; RRID: Addgene_12260) and pMDLg/pRRE (Addgene plasmid 12251; http://n2t.net/addgene:12251; RRID: Addgene_12251) were gifts from Didier Trono. After 24 hours, 3 ml of DMEM was added to the cells, and, after 72 hours of incubation, lentivirus was harvested. We filtered the pooled lentivirus through a 0.45-μm PVDF filter (Millipore) to remove any cellular debris.

To create polyclonal landing pad cell lines, 2 ml of lentiviral supernatant and 8 μg ml^−1^ of polybrene was used on 3 × 10^5^ K562 cells to ensure a high MOI. These cells were infected by spinfection for 30 minutes at 1,000*g* at 33 °C, followed by overnight infection. The next day, the cells were spun down and resuspended in fresh media. This resulted in >50% EGFP^+^ cell populations, suggesting that each cell likely contained multiple landing pad copies. To create clonal landing pad cell lines, lentivirus doses of 50 μl, 100 μl and 200 μl were used for each vector, to find a condition with low MOI wherein each transduced cell would be likely to contain only a single integrated copy of the landing pad. In total, 3 × 10^5^ K562 cells were mixed with the lentiviruses in 8 μg ml^−1^ of polybrene and infected overnight, without spinfection. Infected cells grew for 3 days, and then infection efficiency was measured using flow cytometry to measure EGFP (BD Accuri C6); the dose that gave rise to 5–15% EGFP^+^ cells was selected for each LSR for further experiments. Ten days later, these EGFP^+^ cells were sorted into a 96-well plate with a single cell in each well, to derive clonal lines with a single landing pad location. Two weeks later, 16 wells per LSR were analyzed by flow cytometry (BD Accuri C6). In some cases, the well was empty, possibly due to a failure to sort a single cell into that well or because the cells died. Four clones for each LSR with a unimodal high EGFP expression level were selected for expansion and subsequent experiments. Altogether, 27 days passed from infection to clone selection, so these are clones that show high EGFP expression stability.

### Landing pad integration efficiency assay

Landing pad cell lines were electroporated in 100 μl of Amaxa solution (Lonza Nucleofector 2b, program T-016) with the promoterless mCherry donor containing the matching attP at a dose of either 1,000 ng or 3,000 ng of donor plasmid using 400,000 cells per electroporation. At timepoints from 5 days to 12 days after electroporation, the cells were subjected to flow cytometry to measure mCherry and EGFP (BD Accuri C6 or Bio-Rad ZE5).

### Pseudosite integration efficiency assay to measure integration into the human genome

To determine the efficiency of integration of attD donors into pseudosites in the human genome, attD sequences were cloned into a plasmid containing a EF-1α promoter, followed by mCherry, a P2A self-cleaving peptide and a puromycin resistance marker. Integration efficiency was measured in both K562 and HEK293FT cells. In K562 cells, 1.0 × 10^6^ cells were electroporated in 100 μl of Amaxa solution (Lonza Nucleofector SF, program FF-120), with 3,000 ng of LSR plasmid and 2,000 ng of pseudosite attD plasmid. As a non-matching LSR control, 3,000 ng of Bxb1 was substituted for the correct LSR plasmid. A similar experiment was performed with additional doses (1,000–3,000 ng) for Cp36 LSR plasmid, and the attD donor plasmid was delivered at a 1:1 molar ratio. The cells were cultured between 2 × 10^5^ cells per milliliter and 1 × 10^6^ cells per milliliter for 2–3 weeks.

In HEK293FT cells, 20,000 cells were plated into 96-well plates and transfected 1 day later with 200 ng of LSR plasmid and 178 ng of pseudosite attD plasmid using Lipofectamine 2000 (Invitrogen). As a non-matching LSR control, 200 ng of Bxb1 was substituted for the correct LSR plasmid. Additionally, a linear version of the pseudosite attD donor was also tested for integration activity in HEK293FT cells. To create the linear donors, pseudosite attD plasmids were PCR amplified using the KAPA Hifi HotStart ReadyMix (Roche), amplifying the attD and the EF-1α promoter, followed by mCherry, a P2A self-cleaving peptide and a puromycin resistance marker. The PCR product was gel extracted with the Monarch DNA Gel Extraction Kit (New England Biolabs (NEB)). In total, 20,000 HEK293FT cells were plated into 96-well plates and transfected 1 day later with 300 ng of LSR plasmid and 24 ng of the linear pseudosite attD donor. As a non-matching LSR control, 300 ng of Bxb1 was substituted for the correct LSR plasmid.

For all K562 and HEK293FT transfections, 100 μl of each sample was run on the Attune NxT Flow Cytometer every 3–4 days to measure the mCherry signal. After 2–3 weeks, transiently transfected plasmid was nearly fully diluted out in the non-matching LSR control, and the efficiency of the LSR was determined by the difference in percentage of mCherry^+^ cells between the non-matching LSR control and the experimental condition.

### Generation of donor plasmids containing unique molecular identifiers

To differentiate unique integration events from clonal expansion or PCR duplicates, unique molecular identifiers (UMIs) were cloned into the pEF-1α-mCherry-P2A-Puro donor plasmids. Nx12 oligos were synthesized by Integrated DNA Technologies (IDT); six-cycle PCR using Kapa Hifi PCR Mastermix (Roche) was performed with BsaI Golden Gate overhangs to create a double-stranded UMI library insert; and the PCR was purified using DNA Clean and Concentrator-5 (Zymo). Next, the UMI library was assembled via a Golden Gate reaction into a BsaI landing pad located upstream of the EF-1α promoter. The Golden Gate reaction specifications are as follows: 127 ng of purified insert; 2 μg of pre-digested (BsaI) and purified (DNA Clean and Concentrator-5, Zymo) backbone (3:1 ratio of insert to backbone); 5 μl of 10× T4 DNA Ligase Buffer (NEB); 2.5 μl of T4 DNA Ligase (NEB); 2.5 μl of BsaI-HFv2 (NEB); and water to a final volume of 50 μl. The reaction was run for 1 hour at 37 °C and then inactivated for 20 minutes at 80 °C. The sample was then purified using DNA Clean and Concentrator-5 (Zymo) with the specified plasmid protocol and quantified via NanoDrop. Then, 1 μl of the library was electroporated into Endura Electrocompetent Cells (Lucigen) using the recommended optional electroporation protocol, plated onto two 500-cm^2^ BioAssay plates, grown at 30 °C for 16 hours and harvested using the NucleoBond Xtra Maxi EF Kit (Macherey-Nagel). UMI coverage was calculated via dilution plating, which was determined to be 144× coverage of the 16 million UMIs. attD sequences for each LSR were next cloned into these UMI-containing backbones, using Golden Gate assembly into a Esp3I landing pad directly upstream of the UMI. Each assembly contained 34 ng of purified attD insert with Esp3I overhangs, 521 ng of pre-digested, purified backbone (3:1 ratio insert to backbone), 5 ul of 10× T4 DNA Ligase Buffer (NEB), 2.5 ul of T4 DNA Ligase (NEB), 2.5 μl of Esp3I (Thermo Fisher Scientific) and water to a final volume of 50 μl. Then, 1 μl of the library was electroporated into Endura Electrocompetent Cells (Lucigen) using the recommended optional electroporation protocol, seeded directly into liquid culture in Terrific Broth, grown overnight at 37 °C and harvested using the NucleoBond Xtra Maxi EF Kit (Macherey-Nagel). Calculated UMI library coverage was greater than 30× for all donor plasmids.

### Integration site mapping assay to determine human genome integration specificity

Integration site mapping was performed on both K562 and HEK293FT cells. In total, 1.0 × 10^6^ K562 cells were electroporated in Amaxa solution (Lonza Nucleofector SF, program FF-120) with LSR and pseudosite attD plasmids, using the protocol as above for the pseudosite integration efficiency assay. For HEK293FT cells, 20,000 cells were plated into 96-well plates and transfected 1 day later with 200 ng of LSR plasmid and 178 ng of pseudosite attD plasmid using Lipofectamine 2000 (Invitrogen). After 5 days in culture, puromycin was added to the media at 1 μg ml^−1^ for K562 cells and 0.5 μg ml^−1^ for HEK293FT cells. The cells were cultured for two more weeks, and then the gDNA was harvested using the Quick-DNA Miniprep Kit (Zymo) and quantified by Qubit HS dsDNA Assay (Thermo Fisher Scientific). A modified version of the UDiTaS sequencing assay was then used as described below^35,36^. Tn5 was purified using the protocol described in Picelli et al.^95^ and stored at 7.5 mg ml^−1^. Adaptors were assembled by combining 50 μl of 100 μM top and bottom strand, heating to 95 °C for 2 minutes and slowly ramping down to 25 °C over 12 hours. Next, the transposome was assembled by combining 85.7 μl of Tn5 transposase with 14.3 μl of pre-annealed oligos and incubated for 60 minutes at room temperature. Tagmentation was performed by adding 150 ng of gDNA, 4 μl of 5× TAPS-DMF (50 mM TAPS NaOH, 25 mM MgCl_2_, 50% v/v DMF (pH 8.5) at 25 °C), 3 μl of assembled transposome and water for a final reaction volume of 20 μl. The reaction was incubated at 55 °C for 10–15 minutes and then purified with Zymo DNA Clean and Concentrator-5. The tagmented products were run on Agilent Bioanalyzer HS DNA Kit to confirm average fragment size of ~2 kb. Next, PCR was performed with the outer primers (P5_outer, pseudosite_donor_outer; Supplementary Table 6) for 12 cycles using 12.5 μl of Platinum Superfi PCR Master Mix (Thermo Fisher Scientific), 1.5 μl of 0.5 M TMAC, 0.5 μl of 10 μM pseudosite_donor_outer primer, 0.25 μl of 10 μM P5_outer primer, 9 μl of tagmented DNA and 1.25 μl of DMSO. After AMPure XP 0.9× bead cleanup, a second PCR with the inner nested primers (P5_inner, i7 primers; Supplementary Table 6) was performed for 18 cycles. The PCR contained 25 μl of Platinum Superfi Master Mix (Thermo Fisher Scientific), 3 μl of 0.5 M TMAC, 2.5 μl of DMSO, 2.5 μl of 10 μM P5_inner primer, 5 μl of 10 μM i7 primer, 10 μl of the purified 1st round PCR product and 2 μl of water for a final reaction volume of 50 μl. The final library was size selected on a 2% agarose gel for fragments between 300 bp and 800 bp, gel extracted with the Monarch DNA Gel Extraction Kit (NEB), quantified with Qubit HS dsDNA Assay (Thermo Fisher Scientific) and KAPA Library Quantification Kit (Roche), fragment analyzed with Agilent Bioanalyzer HS DNA Kit and sequenced on a MiSeq (Illumina MiSeq Control Software version 4.0.0.1769). The same protocol was performed for on-target and off-target integration mapping on the landing pad samples, with different donor outer and i7 primers corresponding to the donor plasmid used (LP_donor_outer, LP_i7 primers; Supplementary Table 6).

### Computational analysis of integration site mapping assay

Snakemake (version 5.32.0) workflows were constructed and used to analyze NGS data from the integration site mapping assay^96^. First, stagger sequences added to primers during library preparation were removed using custom Python scripts. Next, fastp (version 0.19.6) was used to trim Nextera adapters from reads and to remove reads with low PHRED scores^97^. Next, reads were aligned to both the human genome (GRCh38) and a donor plasmid sequence containing the LSR-specific attD sequence in single-end mode using BWA MEM (version 0.7.17)^98^. Next, reads were analyzed individually using custom Python scripts to identify (1) if they aligned to the donor plasmid, human genome or both; (2) whether or not the reads began at the predicted primer; (3) whether or not the pre-integration attachment site was intact; and (4) whether or not the attachment site matched the expected donor plasmid. Reads were then filtered to include only those reads that mapped to both the donor plasmid and the human genome, those that began at the primer site and those that did not have an intact attD sequence (if this could be determined from the length of a particular read). This filtered read set was then aligned in paired-end mode to the human genome using default settings in BWA MEM. Alignments with a mapping quality score less than 30 were removed, along with supplementary alignments and paired read alignments with an insert size longer than 1,500 bp. The SAMtools markdup tool was used to remove potential PCR duplicates and identify unique reads for downstream analysis^99^. Next, MGEfinder was used to extract clipped-end sequences from reads aligned to the human genome and generate a consensus sequence of the clipped ends, which represent the crossover from the human genome into the integrated attD sequence^27^. Using custom Python scripts, k-mers of length 9 bp were extracted from these consensus sequences and compared with a subsequence of the attD plasmid extending from the original primer to 25 bp after the end of the attD attachment site. If there were no shared 9-mers, the candidate was discarded. Otherwise, consensus sequences were clipped to begin at the primer site, and these consensus sequences were then aligned back to the original attD subsequence using the biopython local alignment tool^100^. Two aligned portions were extracted: the full local alignment of the consensus sequence to the attD (called the ‘full local alignment’) and the longest subset of the alignment that included no ambiguous bases and no gaps (called the ‘contiguous alignment’). To filter a final set of true insertion sites, only sites with at least 80% nucleotide identity shared between the consensus sequence and the attD subsequence in either the full local alignment or the contiguous alignment were kept. Finally, only sites with a crossover point within 15 bp of the predicted dinucleotide core were kept.

This approach could precisely predict integration sites, but errors in sequencing reads led to some variability in this prediction. To account for this, integration sites were combined into integration ‘loci’ by merging all sites that were within 500 bp of each other, using bedtools (version 2.27.0)^101^. This approach would merge integration events that occurred at the same site but in opposite orientations, for example. When pooling reads across biological or technical replicates, these loci were also merged if they overlapped. When measuring the relative frequency of insertion across different loci, all uniquely aligned reads (de-duplicated using SAMtools markdup) found within each locus were counted, or UMIs were counted if they were available. These were then converted into percentages for each locus by dividing by the total number of unique reads/UMIs aligned to all integration loci.

Target site motifs for different LSRs could be determined from precise predictions of dinucleotide cores for all integration sites. For each integration locus, only one integration site was chosen if there were multiple, and integration sites with more reads supporting them were prioritized. Human genome sequences around the predicted dinucleotide core were extracted using bedtools, choosing the forward or reverse strand depending on the orientation of the integration. All such target sites, or a subset of these target sites if desired, were then analyzed for conservation at each nucleotide position using the ggseqlogo package in R (ref. ^83^).

### DNase hypersensitivity integration site enrichment for multi-targeting LSRs

ENCODE DNase hypersensitivity regions (also referred to as peaks) were used to identify integration sites that overlapped with regions of accessible chromatin. For K562 cells, the DNase hypersensitivity peaks identified in experiment ENCFF274YGF were used, and, for HEK293T (HEK293FT peaks were not available), the peaks identified in experiment ENCFF274YGF were used^102,103^. Enrichment of integration sites within DNase hypersensitivity sites was calculated using a two-sided Fisher’s exact test, with random background sites selected by randomly choosing two sites from that were within 100 kbp of each true integration site.

### Post hoc identification of human genome integration sites using database-derived motifs

A computational approach was designed that started with a query LSR sequence and then built sequence motifs by iteratively adding natural attB sequences of the next most closely related LSR ortholog, only adding additional attB sequences if they were 95% identical or less to already selected attB sequences. Only attB sequences that belonged to relatives that were at least 30% identical at the amino acid level to the queried LSR were considered. The attB sequences were oriented with respect to each other by choosing the strand orientation with the highest global alignment to the query LSR’s attB sequence. All attB sequences were then aligned together using MUSCLE to generate a multiple sequence alignment (MSA). The middle 60 nucleotides of the MSA were then extracted, excluding columns with over 50% gaps, and the nucleotide frequencies were mapped onto the query LSR’s attB sequence to generate a final motif, with gaps being replaced by nucleotides with equally weighted frequencies. Motifs built from 20, 50 and 100 such attB sequences were constructed. Then, motifs were searched against the experimentally observed human integration sites and approximately 30,000 randomly selected human genome sequences using HOMER with no minimum score threshold^104^. HOMER calculates motif scores for each searched sequence by taking the sum of the log-odds probabilities at each nucleotide position. Next, R scripts were used to iterate across a range of motif score cutoffs to calculate the true-positive rate and the false-positive rate at each cutoff, generating a ROC curve. For each LSR, the motif with the greatest AUC was selected from the three motifs that were constructed.

### NGS of linear donor recircularization and integration

Linear donors were generated as described above (in the section titled ‘Pseudosite integration efficiency assay to measure integration into the human genome’), resulting in a purified PCR product containing the attD and the EF-1α promoter, followed by mCherry, a P2A self-cleaving peptide and a puromycin resistance marker. In total, 20,000 HEK293FT cells were plated into a 96-well plate and 1 day later transfected with 24 ng of linear donor and 300 ng of cognate LSR or Bxb1 as a non-matching LSR control. After 5 days, puromycin was added to enrich for integrants. The cells were cultured for two more weeks, and then gDNA was extracted using the Quick-DNA Miniprep Kit (Zymo) and quantified by Qubit HS dsDNA Assay (Thermo Fisher Scientific). PCR primers (Linear_donor_jxn_F, Linear_donor_jxn_R; Supplementary Table 6) were designed to specifically amplify outwards from the ends of the linear donor, and PCR was performed on the linear donor DNA alone and on the DNA extracted from transfections containing the linear donor with and without cognate LSR. PCR products were only visible by gel electrophoresis when using the post-transfection template DNA. PCR products were amplified with Flap2 primers (Supplementary Table 6) to add P5 and P7 adaptors for illumina sequencing and sequenced with the Illumina MiSeq v2 2 × 150 paired-end reads. To perform indel analysis, sequencing was run through the CRISPResso2 (version 2.0.20b) workflow^105^, with custom parameters: the amplicon was set to be a concatenation of the right and then left flanks of the linear donor; the single guide RNA (sgRNA) was set to be the sequence directly 5′ of the expected rejoining site of the two linear donor ends; the quantification window was set to 0 (relative to the 3′ end of the sgRNA); and the quantification window size was set to 1.

### Comparison of LSR and PiggyBac transposase efficiency

In total, 1.2 × 10^6^ K562 cells were electroporated in 100 μl of Amaxa solution (Lonza Nucleofector 2b, program T-016) with 2,000 ng of a pEF-1α-PuroR-P2A-mCherry donor plasmid containing an upstream Cp36 attD site (pJT371), in combination with 3,000 ng of Cp36 expression vector. Cells were grown for 10 days and then analyzed using flow cytometry for mCherry fluorescence (Bio-Rad ZE5, Everest software version 3.1) with analysis using CytoFlow (https://github.com/cytoflow/cytoflow).

### Assessment of Cp36 directionality via redosing

To test for possible excision upon Cp36 re-dosing, 1.0 × 10^6^ K562 cells were electroporated in 100 μl of Amaxa solution (Lonza Nucleofector SF, program FF-120) with 3,000 ng of Cp36 LSR plasmid and 2,000 ng of the Cp36 pseudosite attD plasmid with an mCherry expression cassette or attD plasmid alone. After 15 days, the Cp36-treated cells were re-electroporated with 3,000 ng of Cp36 LSR plasmid or empty LSR backbone control plasmid. Three days later, the cells were measured by flow cytometry (Attune NxT) for mCherry fluorescence.

To generate a pure population of stable mCherry-integrated cells using Cp36, 1.2 × 10^6^ K562 cells were electroporated in 100 μl of Amaxa solution (Lonza Nucleofector 2b, program T-016) with 2,000 ng of the same Cp36 PuroR-P2A-mCherry donor, in combination with 3,000 ng of Cp36 expression vector. After 3 weeks of growth to allow the donor plasmid to dilute, cells with integrants were selected to purity using 1 μg ml^−1^ of puromycin over 7 days and confirmed using flow cytometry for mCherry fluorescence (Attune NxT). To assess the efficiency of integrating a second donor sequence, we generated a second fluorescent donor construct (pJT396) by replacing mCherry in pJT371 with mTagBFP2 and prepared DNA by Mira prep^106^. We then electroporated 4.0 × 10^5^ of wild-type or the stably integrated mCherry K562 cell lines in 100 μl of Amaxa solution (Lonza Nucleofector 2b, program T-016) with pJT396 in combination with an equimolar amount of either pUC19 or a Cp36 expression vector, totalling approximately 4 μg of DNA. The frequency of doubly integrated cells was assessed using flow cytometry for mCherry and mTagBFP2 fluorescence at 13 days after electroporation (Attune NxT), with analysis performed in FlowJo. Note that this method differs from that used for mCherry in the initial pseudosite integration assay.

### Simultaneous stable delivery of two genes with Cp36

To generate stable mCherry-integrated and BFP-integrated cells using Cp36, 1.2 × 10^6^ K562 cells were electroporated in 100 μl of Amaxa solution (Lonza Nucleofector 2b, program T-016) with 3,000 ng of both the same Cp36 PuroR-P2A-mCherry donor and Cp36 PuroR-P2A-mTagBFP2 donor, in combination with an equimolar dose of 2,400 ng of Cp36 expression vector. Control cells were treated with pUC19 and donors, Cp36 and pUC19 or a single donor and Cp36 or pUC19. The frequency of singly and doubly integrated cells was assessed using flow cytometry for mCherry and mTagBFP2 fluorescence (Attune NxT), with analysis performed in FlowJo.

### Activity assay of synthetic enhancer reporters installed at AAVS1

To install the synthetic transcription factor rTetR-VP48 into wild-type K562 cells, 1.0 × 10^6^ wild-type K562 cells were electroporated in 100 μl of Amaxa solution (Lonza Nucleofector 2b, program T-016) with 1 μg of PiggyBac expression vector (PB200A-1, SBI) and 1 μg of pMMH4, an ITR-flanked plasmid harboring the EF-1α core promoter driving rTetR-VP48-T2A-hygromycin resistance gene and a separate Tet responsive promoter (TRE3G) driving an mCherry gene. Integrants were selected to purity using 200 μg ml^−1^ of hygromycin (Thermo Fisher Scientific) over 7 days. Enhancer reporter donor constructs flanked by AAVS1 homology arms (pMMH23,24,26) were subsequently integrated into the AAVS1 locus of cells expressing rTetR-VP48 using TALEN-mediated HDR as follows: 1.0 × 10^6^ K562 cells were electroporated in Amaxa solution (Lonza Nucleofector 2b, setting T0–16) with 1,000 ng of reporter and 500 ng of each TALEN-L (Addgene, 35431) and TALEN-R (Addgene, 35432) plasmid (targeting upstream and downstream the intended DNA cleavage site, respectively). In the pooled reporter assay, a small library of Tet responsive elements was ordered as an oligo pool (opJS2, IDT), assembled into the reporter plasmid, mini-prepped and electroporated as a pool. The reporters contain a promoterless puromycin resistance gene that traps the AAVS1 promoter. Two days after electroporation, the cells were treated with 1 μg ml^−1^ of puromycin antibiotic for 7 days to select for a population with reporter donor integrated into AAVS1. Reporter expression was measured by flow cytometry (Bio-Rad ZE5) after 2 days of 1,000 ng ml^−1^ doxycycline induction (Thermo Fisher Scientific).

### Activity assay of synthetic enhancer reporters installed at a landing pad

To install the synthetic transcription factor rTetR-VP48 into landing pad cells, 1.0 × 10^6^ clonal Kp03 landing pad cells were electroporated in 100 μl of Amaxa solution (Lonza Nucleofector 2b, program T-016) with 1 μg of PiggyBac expression vector (PB200A-1, SBI) and 1 μg of pMMH4 and selected to purity using 200 μg ml^−1^ of hygromycin (Thermo Fisher Scientific) over 7 days. To install enhancer reporter plasmids at the landing pad, 1.0 × 10^6^ K562 cells harboring a monoclonal Kp03 landing pad and multiclonal rTetR-VP48 expression construct were electroporated in 100 μl of Amaxa solution (Lonza Nucleofector 2b, program T-016) with 1,000 ng of reporter donor plasmid (pMMH56,59,59). In the pooled reporter assay, 200 ng of each of five reporter constructs (pMMH55–59) were combined and electroporated together. As a negative control, cells were electroporated with 1,000 ng of reporter donor with no attP site upstream of the promoterless puro resistance gene. The reporters contain a promoterless puromycin resistance gene that traps the landing pad promoter. Three days after electroporation, the cells were treated with 1 ng ml^−1^ of puromycin antibiotic for 7 days to select for a population with reporter donor correctly integrated into the landing pad. All negative control cells died during selection. Reporter expression was measured at the end of selection by flow cytometry (Bio-Rad ZE5) after 2 days of 1,000 ng ml^−1^ doxycycline induction (Thermo Fisher Scientific).

### Magnetic separation of cells based on reporter expression level

The reporter included a synthetic surface marker, consisting of the human IgG1 Fc region linked to an Igκ leader and PDGFRb transmembrane domain, to enable magnetic separation of OFF from ON cells, which we previously used to study transcriptional effector domains^46^ and here adapted to study enhancers. Before magnetic separation, the cells were cultured between 2 × 10^5^ cells per milliliter and 1 × 10^6^ cells per milliliter for 2 weeks after selection. After 2 days of 1,000 ng ml^−1^ doxycycline induction, 1 × 10^7^ cells were spun down at 300*g* for 5 minutes, and media was aspirated. Cells were then resuspended in the same volume of PBS (Gibco), and the spin-down and aspiration was repeated to wash the cells and remove any IgG from serum. Dynabeads M-280 Protein G (Thermo Fisher Scientific, 10003D) were resuspended by vortexing for 30 seconds. Then, 50 ml of blocking buffer was prepared by adding 1 g of biotin-free BSA (Sigma-Aldrich) and 200 μl of 0.5 M pH 8.0 EDTA (Thermo Fisher Scientific, 15575020) into DPBS (Gibco), vacuum filtering with a 0.22-μm filter (Millipore) and then kept on ice. Next, 50 μl of beads was prepared for every 1 × 10^7^ cells by adding 1 ml of buffer per 200 μl of beads, vortexing for 5 seconds, placing on a magnetic tube rack (Eppendorf), waiting 1 minute, removing the supernatant and finally removing the beads from the magnet and resuspending in 100–600 μl of blocking buffer per initial 50 μl of beads. Beads were added to cells at 1 × 10^7^ cells per 25 μl of resuspended beads and then incubated at room temperature while rocking for 30 minutes. We used non-stick Ambion 1.5-ml tubes and a small magnetic rack. After incubation, the bead and cell mixture was placed on the magnetic rack for >2 minutes. The unbound supernatant was transferred to a new tube and placed on the magnet again for >2 minutes to remove any remaining beads, and then the supernatant was transferred to a new tube. For the LSR PRA, the same magnetic separation procedure was performed two more times (for a total of three times) on this supernatant to remove cells with activated reporters from the unbound population. Only the final unbound population was saved for further analysis by flow cytometry and library preparation. The beads from the first round of magnetic separation were resuspended in the same volume of blocking buffer and magnetically separated again, and then the supernatant was discarded. Resuspension, magnetic separation and discarding the supernatant was repeated, and the tube with the beads was kept as the bound fraction. The bound fraction was resuspended in blocking buffer or PBS to dilute the cells (the unbound fraction is already dilute). Flow cytometry (Bio-Rad ZE5) was performed using a small portion of each fraction to estimate the number of cells in each fraction and to confirm separation based on reporter levels. Finally, the samples were spun down, and the pellets were frozen at 20 °C until gDNA extraction. Two additional biological replicates of the LSR PRA were performed similarly at a later date, starting from the step of electroporating cells with the pooled reporter plasmid donors.

### Library preparation and sequencing of magnetically separated reporter cell pool

gDNA was extracted using Monarch Genomic DNA Purification Kit (NEB) according to manufacturer instructions. After cell lysis, magnetic separation was performed on the bound population to remove beads. No more than 5 × 10^6^ cells were loaded onto a single column and eluted with water to avoid subsequent PCR inhibition. Libraries were assembled using three PCRs: PCR1 amplifies enhancer elements off the genome; PCR2 extends these amplicons with TruSeq R1/R2 handle sequences; and PCR3 extends these amplicons to add sample barcodes and p5/p7 sequences. PCR1 reactions contained 20 μl of purified gDNA, 2.5 μl of each 10 μM primer (cTF98 and cTF109; Supplementary Table 6) and 25 μl of Q5 2× Master Mix (NEB) and was amplified with the following thermocycling conditions: 3 minutes at 98 °C and then 23× cycles of 10 seconds at 98 °C, 30 seconds at 66 °C and 1 minute at 72 °C and then a final extension step of 72 °C for 5 minutes. The PCR product was purified using 45 μl of SPRI beads (Beckman Coulter) (0.9× of PCR volume) according to manufacturer instructions and eluted in 21 μl of nuclease-free water. PCR2 reactions were assembled with 1 μl of purified PCR1 product, 1 μl of each 10 μM primer (oBD55 and oBD68), 10 μl of Q5 2× Master Mix and 7 μl of nuclease-free water and amplified using the following thermocycling conditions: 30 seconds at 98 °C and then 3–7× cycles of 10 seconds at 98 °C, 30 seconds at 68 °C, 20 seconds at 72 °C and then a final step of 72 °C for 5 minutes. The PCR2 product was purified using 18 μl of SPRI beads (0.9× of PCR volume) according to manufacturer instructions and eluted in 21 μl of nuclease-free water. PCR3 reactions contained 1 μl of purified PCR2 product, 1 μl of each 10 μM primer (oBD19–26), 10 μl of Q5 2× Master Mix and 7 μl of nuclease-free water. The same thermocycling and purification protocol from PCR2 was performed. Purified PCR3 products were confirmed to be the correct size using a D1000 TapeStation (Agilent) and quantified with a Qubit HS kit. Samples were pooled with PhiX (Illumina) to ensure appropriate library complexity and sequenced on an Illumina MiSeq with a Nano kit with 4–8 indexing cycles and 150 cycle paired-end reads.

### Analysis of PRA sequencing data

Sequencing reads were demultiplexed using bcl2fastq (version 2.20). The HT-recruit-Analyze processing pipeline was used to generate a Bowtie reference and modified to align paired-end reads with 0 mismatch allowance (https://github.com/bintulab/HT-recruit-Analyze). Count matrices for the bound and unbound samples were then used to calculate log_2_(ON:OFF) for each enhancer, normalizing for read depth across bound and unbound samples.

### Amplicon barcode library installation

A library of mCherry amplicons with randomized barcodes after the stop codon was generated by PCR, electroporated into landing pad cells and recovered and sequenced from gDNA. More specifically, to construct the library, primers were designed to amplify the attP-mCherry-pA sequence off of the template plasmids used in previous landing pad assays (pC432 and pC494; Supplementary Table 6), and the reverse primer included a randomized 6×N barcode as an extension. This primer was synthesized by IDT using standard mixed bases. A mastermix for 8× reactions of PCR was made using 80 ng of plasmid template, 200 μl of 2× Q5 MM (NEB), 10 μl each of 100 nM forward and reverse primers (cTF334, JT1046) and 172 μl of nuclease-free water and then split into separate reactions and amplified with the following thermocycling conditions: 2 minutes at 98 °C and then 30× cycles of 10 seconds at 98 °C, 30 seconds at 65 °C and 40 seconds at 72 °C and then a final extension step of 72 °C for 5 minutes. The length of the library was confirmed by gel electrophoresis, and its concentration was measured by NanoDrop.

K562 landing pad clonal lines with the associated (or mismatched, as a control) recombinase were then electroporated with these amplicon donors. In total, 1.2 × 10^6^ K562 cells were electroporated in 100 μl of Amaxa solution (Lonza Nucleofector 2b, program T-016) with either 250 ng or 750 ng of the amplicon donor. Seven days later, the efficiency of mCherry integration was determined by flow cytometry (Bio-Rad ZE5), and then, 1 day later, gDNA extraction was performed from 5 million cells using a Qiagen DNeasy Mini Prep Kit.

Another experiment was performed similarly with other doses. In total, 1.2 × 10^6^ K562 cells were electroporated in 100 μl of Amaxa solution (Lonza Nucleofector 2b, program T-016) with either 500 ng or 2,000 ng of amplicon donors. We also included a matched plasmid donor condition: 4,615 ng of plasmid was used to provide an equimolar dose as 2,000 ng of amplicon donor. Each donor condition was tested in two different clonal Kp03 landing pad lines. Six days later, the efficiency of mCherry integration was determined by flow cytometry (Bio-Rad ZE5).

### Junction PCR library preparation and sequencing of amplicon donor barcodes

NGS libraries were prepared from the extracted gDNA harvested 8 days after electroporation of landing pad cells with 750 ng of a 1.2-kb attP-mCherry-pA-Barcode amplicon. Libraries were assembled using three rounds of PCR that only captures barcodes successfully integrated into the on-target landing pad site in the genome: PCR1 amplifies barcodes off the genome across the 3′ donor–genome junction; nested PCR2 further amplifies the barcodes and extends them with TruSeq R1/R2 handle sequences; and PCR3 extends the amplicons to add sample indices and p5/p7 sequences. Specifically, PCR1 mastermixes were assembled with 480 μl of gDNA, 500 μl of 2× NEBNext Ultra II Master Mix (NEB) and 10 μl each of 100 nM forward and reverse primers (cTF347 is a universal forward primer, and JT1067/8 are reverse primers specific to the associated landing pad; Supplementary Table 6) and then split into ten separate 100-μl reactions and amplified with the following thermocycling conditions: 1 minute at 98 °C and then 35× cycles of 10 seconds at 98 °C, 30 seconds at 68 °C and 45 seconds at 65 °C and then a final extension step of 65 °C for 5 minutes. Gel electrophoresis of junction PCR with matched and mismatched LSR donor samples was performed to confirm that the PCR1 product was specific to cells with on-target integrations. Then, 50 μl of the junction PCR1 product was purified using 45 μl of SPRI beads (Beckman Coulter) (0.9× of PCR volume) according to manufacturer instructions and eluted in 23.5 μl of nuclease-free water. PCR2 reactions were assembled with 22.5 μl of purified PCR1 product, 2.5 μl of pooled 10 μM forward and reverse primer (cTF348 and cTF351) and 25 μl of 2× NEBNext Ultra II Master Mix (NEB) and then thermocycled as follows: 30 seconds at 98 °C and then 6× cycles of 10 seconds at 98 °C, 30 seconds at 68 °C, 20 seconds at 72 °C and then a final step of 72 °C for 5 minutes. The PCR2 product was purified using 45 μl of SPRI beads (0.9× of PCR volume) according to manufacturer instructions and eluted in 21 μl of nuclease free water. PCR3 reactions contained 22.5 μl of purified PCR2 product, 2.5 μl of each 10 μM primer (oBD19–26) and 10 μl of 2× NEBNext Ultra II Master Mix (NEB). The same thermocycling and purification protocol from PCR2 was performed. Purified PCR3 products were confirmed to be the correct size using a D1000 TapeStation (Agilent) and quantified with a Qubit HS kit. Samples were pooled with PhiX (Illumina) to ensure appropriate library complexity and sequenced on an Illumina MiSeq with a Nano kit with 4–8 indexing cycles and 150 cycle paired-end reads.

### Analysis of amplicon donor barcode sequencing data

Sequencing reads were analyzed with a custom Python script to count barcodes. Reads were filtered for an average Qscore ≥30 over all positions and a minimum Qscore ≥30 over the 6-bp barcode region. Matches in that region to any of the 4,096 possible 6×N barcodes were tallied. A barcode was defined as a dropout if there were only 0 or 1 counts. The read depth was 216–272× for the pre-installation control samples and 290–357× for all genomic samples.

## Supplementary Material

Supplementary table 6

Supplementary table 5

Supplementary table 4

Supplementary table 3

Supplementary table 2

Supplementary table 1

Supplementary information

## Data Availability

Publicly available RefSeq and GenBank genomes were used to generate the LSR database. Data to support the results are in the main text and the Supplementary Information. All data to support the results are in the main text, figures and the supplementary tables. Illumina sequencing datasets generated in this study are available on the NCBI Sequence Read Archive, BioProject PRJNA778877 (ref. ^107^).
