# Supplementary material for: Systematic discovery of recombinases for efficient integration of large DNA sequences into the human genome
Source: Nat Biotechnol. 2022 Oct 10;41(4):488–99. doi: 10.1038/s41587-022-01494-w (PMC10083194; doi:10.1038/s41587-022-01494-w)
Supplement: Supplementary file 1 — Supplementary Figs. 1–6, Supplementary Notes 1 and 2 and uncropped gels for Supplementary Fig. 3 [file 41587_2022_1494_MOESM1_ESM.pdf]

nature biotechnology

Article

https://doi.org/10.1038/s41587-022-01494-w

Systematic discovery of recombinases for efficient integration of large DNA sequences into the human genome

In the format provided by the authors and unedited

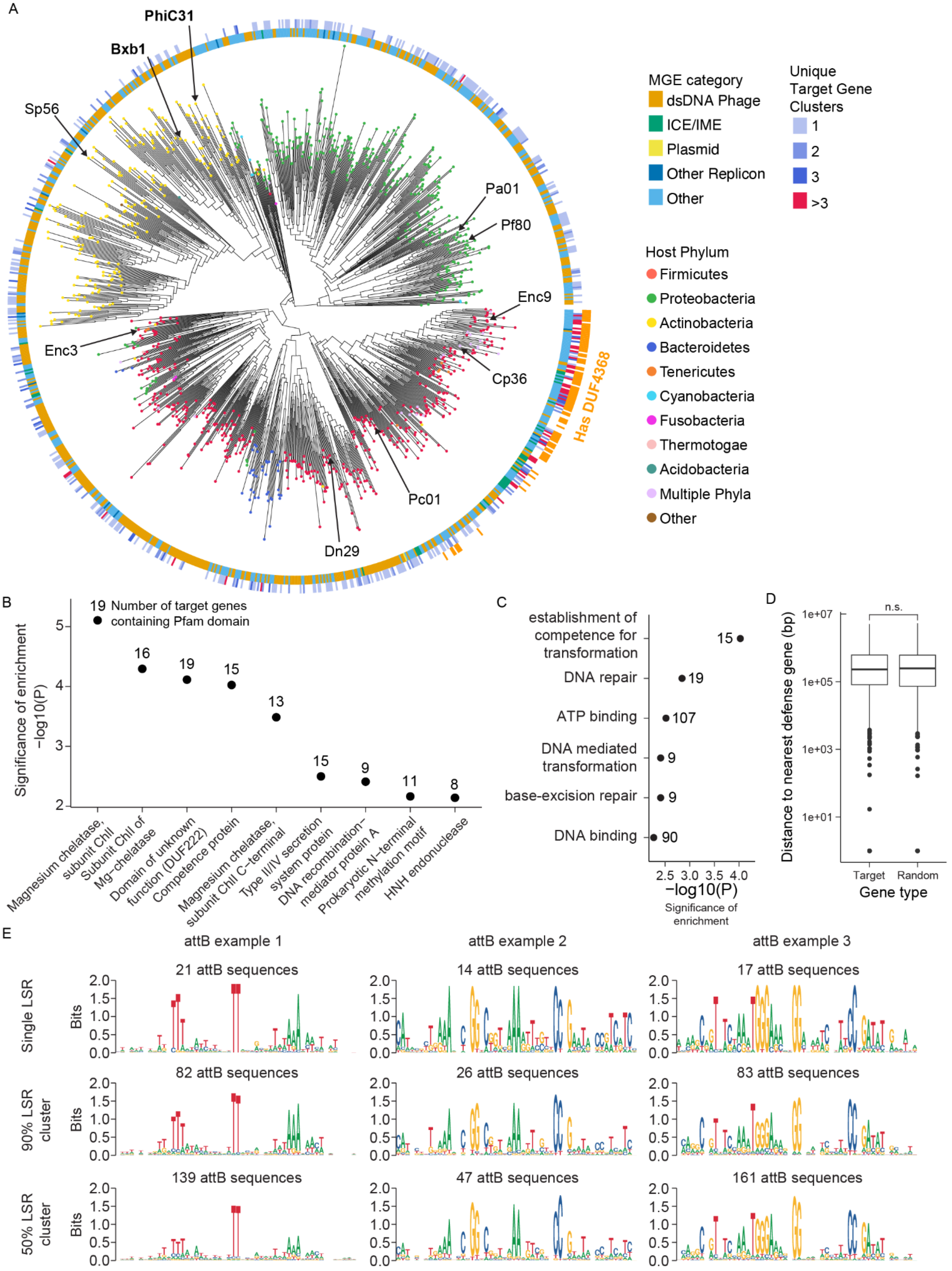

A
PhiC31
Bxb1
Sp56
Pa01
Pf80
Enc9
Enc3
Cp36
Has DUF4368
Pc01
Dn29
MGE category
dsDNA Phage
ICE/IME
Plasmid
Other Replicon
Other
Unique Target Gene Clusters
1
2
3
>3
Host Phylum
Firmicutes
Proteobacteria
Actinobacteria
Bacteroidetes
Tenericutes
Cyanobacteria
Fusobacteria
Thermotogae
Acidobacteria
Multiple Phyla
Other
B
19 Number of target genes containing Pfam domain
Significance of enrichment −log10(P)
Magnesium chelatase, subunit ChlI
Subunit ChlI of Mg-chelatase
Domain of unknown function (DUF222)
Competence protein
Magnesium chelatase, subunit ChlI C-terminal
Type II/IV secretion system protein
DNA recombination-mediator protein A
Prokaryotic N-terminal methylation motif
HNH endonuclease
C
establishment of competence for transformation
DNA repair
ATP binding
DNA mediated transformation
base-excision repair
DNA binding
−log10(P)
Significance of enrichment
D
n.s.
Distance to nearest defense gene (bp)
Target
Random
Gene type
E
attB example 1
attB example 2
attB example 3
21 attB sequences
14 attB sequences
17 attB sequences
82 attB sequences
26 attB sequences
83 attB sequences
139 attB sequences
47 attB sequences
161 attB sequences
Single LSR
90% LSR cluster
50% LSR cluster
Bits

Supplementary Figure 1. Bioinformatic discovery of large serine recombinases and their target attachment sites.

A. Phylogenetic tree of 1,081 LSR clusters (clustered at 50% identity). Tips are colored according to the phylum of bacterial host species. The inner circle of the heat map rings indicates the predicted category of the MGEs that carry a given LSR cluster, with the meaning of the colors indicated in the legend under the title "MGE category." The second layer is colored according to the number of unique target gene clusters that each LSR cluster is predicted to integrate into, as in **Fig. 1B**. The outer ring of orange annotations indicates LSR clusters that are predicted to contain the DUF4368 Pfam domain. Clusters containing Bxb1 and PhiC31 are indicated in bold text, and clusters for select candidates with experimental validation are also indicated. **B.** Pfam domain enrichment analysis of target genes. Pfam domains that reach a significance cutoff of FDR < 0.05 are shown. Pfam domains are ordered and displayed according to the $-\log_{10}(P)$ value of a Fisher's exact test. Numbers next to each point indicate the total number of target gene clusters that contain the specified domain. **C.** Gene ontology (GO) term enrichment analysis of target genes. All 6 terms that reach a significance cutoff of FDR < 0.1 are shown. Terms are ordered and displayed according to the $-\log_{10}(P)$ value of a Fisher's exact test. Numbers next to each point indicate the total number of target gene clusters that fall under the specified GO term. **D.** Distances between target genes and the nearest annotated anti-phage defense gene. For each target gene (n = 808) that appears on a contiguous sequence with a defense gene, the distance is calculated, and then a random gene from the same contiguous sequence is selected as a background control. These data are represented by a boxplot with median, 1st and 3rd quartiles, 1.5 x IQR as whiskers, and outliers as points. A two-sided Wilcoxon rank-sum test was used to test for significant differences between groups (P = 0.75). **E.** Examples of predicted attB motifs. Each column represents a different LSR attB motif. The first row shows motifs that were derived from different attB sequences that were all

targeted by a single, unique LSR protein. The second row shows motifs that were derived from attB sequences that were targeted by LSR proteins that fell into a single 90% identity cluster. The third row shows motifs that were derived from attB sequences that were targeted by LSR proteins that fell into a single 50% identity cluster.

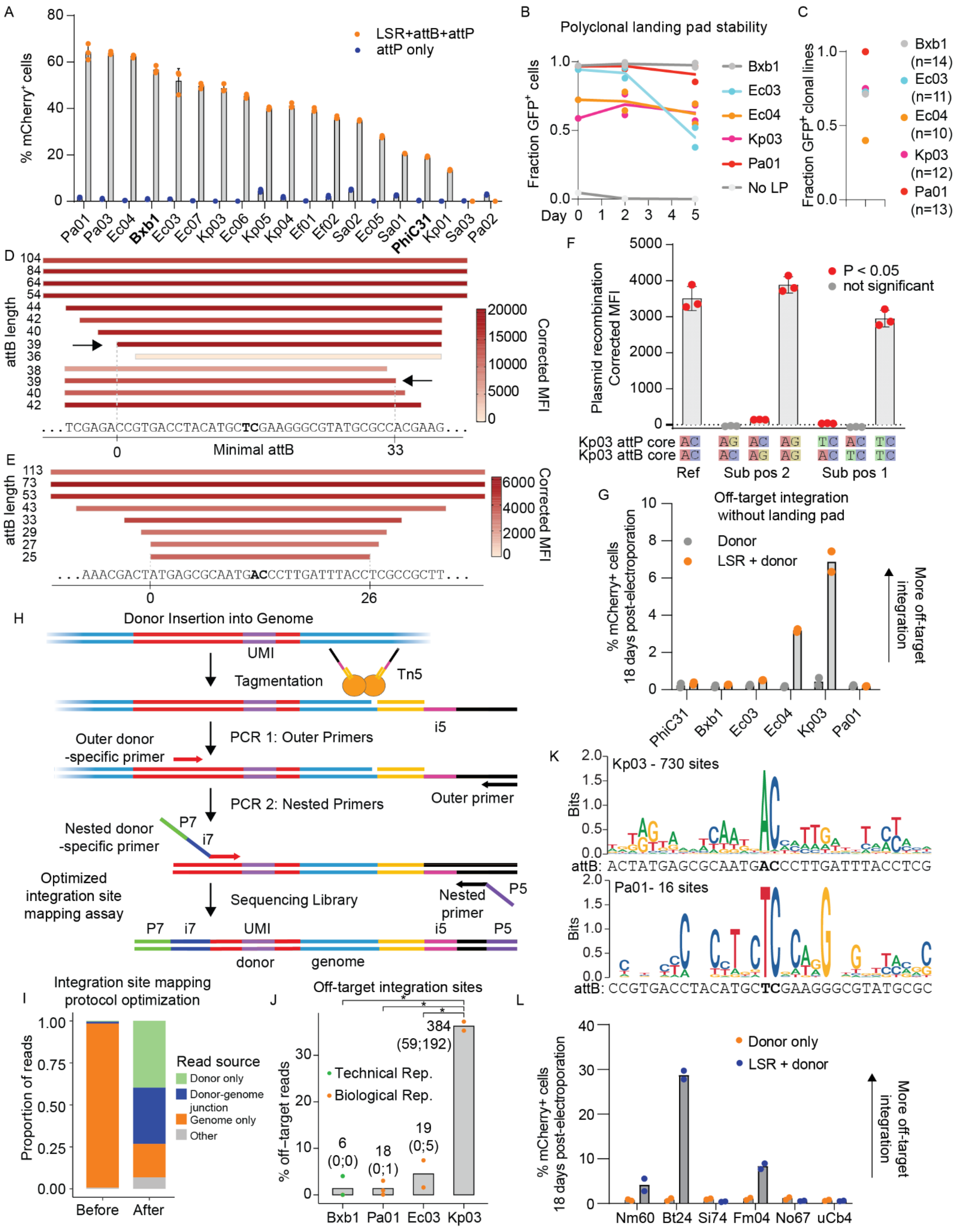
A
% mCherry+ cells
LSR+attB+attP
attP only
Pa01 Pa03 Ec04 Bxb1 Ec03 Ec07 Kp03 Ec06 Kp05 Kp04 Ef01 Ef02 Sa02 Ec05 Sa01 PhiC31 Kp01 Sa03 Pa02
B
Polyclonal landing pad stability
Fraction GFP+ cells
Day
Bxb1
Ec03
Ec04
Kp03
Pa01
No LP
C
Fraction GFP+ clonal lines
Bxb1 (n=14)
Ec03 (n=11)
Ec04 (n=10)
Kp03 (n=12)
Pa01 (n=13)
D
attB length
Corrected MFI
...TCGAGACCGTGACCTACATGCTCGAAGGGCGTATGCGCCACGAAG...
Minimal attB
E
attB length
Corrected MFI
...AAACGACTATGAGCGCAATGACCCTTGATTTACCTCGCCGCTT...
F
Plasmid recombination Corrected MFI
P < 0.05
not significant
Kp03 attP core
Kp03 attB core
Ref
Sub pos 2
Sub pos 1
G
Off-target integration without landing pad
% mCherry+ cells 18 days post-electroporation
Donor
LSR + donor
More off-target integration
PhiC31 Bxb1 Ec03 Ec04 Kp03 Pa01
H
Donor Insertion into Genome
UMI
Tagmentation
Tn5
i5
PCR 1: Outer Primers
Outer donor -specific primer
Outer primer
PCR 2: Nested Primers
Nested donor -specific primer
P7
i7
Optimized integration site mapping assay
Sequencing Library
Nested primer
P5
P7 i7 UMI i5 P5
donor genome
I
Integration site mapping protocol optimization
Proportion of reads
Read source
Donor only
Donor-genome junction
Genome only
Other
Before After
J
Off-target integration sites
% off-target reads
Technical Rep.
Biological Rep.
6 (0;0)
18 (0;1)
19 (0;5)
384 (59;192)
Bxb1 Pa01 Ec03 Kp03
K
Kp03 - 730 sites
Bits
attB: ACTATGAGCGCAATGACCCTTGATTTACCTCG
Pa01- 16 sites
attB: CCGTGACCTACATGCTCGAAGGGCGTATGCGC
L
% mCherry+ cells 18 days post-electroporation
Donor only
LSR + donor
More off-target integration
Nm60 Bt24 Si74 Fm04 No67 uCb4

Supplementary Figure 2. New landing pad LSRs have short attachment sites, can be multiplexed by core swaps, and can be highly specific.

A. Plasmid recombination assay of predicted LSRs and att sites in HEK293FT cells, shown as percentage of mCherry$^+$ cells gated on GFP positive cells. mCherry and GFP gating is determined based on an empty backbone transfection. Dots show each transfection replicate, bars show the mean, error = s.d. (n=3 transfection replicates). **B.** Stability of polyclonal landing pads expressing LSR-GFP as measured by flow cytometry over time. Day 5 was the same day of measurement as for **Fig. 2F.** Lines show the mean. (n=2 independently transduced biological replicates). **C.** Fraction GFP$^+$ cells in clonal cell lines 27 days after transduction. GFP$^+$ cells were sorted into wells as single cells to generate clonal lines, expanded for two weeks, measured by flow cytometry, and graded as GFP$^+$ if the population was >95% GFP$^+$, suggesting a lack of transcriptional silencing. 16 wells were sorted for each LSR, and the number of wells with a live cell population at the time of flow analysis is shown in the legend. For all LSRs, some wells were empty, possibly due to a sorting miss or cell death. **D.** Minimization of Pa01 attB sequence by trimming nucleotides from either end and using the plasmid recombination assay. Arrows indicate shortest attB which did not disrupt recombination activity. The inferred 33 bp minimal attB as determined by this experiment is shown between vertical lines at the bottom. Colored rectangles show mean corrected mCherry MFI (n=3 transfection replicates in HEK293FT cells). The attB in the top rectangle extends in both directions and is the full length attB as retrieved from the LSR database and used in **Fig. 2B and 2C**. A predicted dinucleotide core as determined by off-target integration site mapping is highlighted in bold. **E.** Minimization of Kp03 attB sequence as done in panel D. **F.** Kp03 dinucleotide core substitution in the plasmid recombination assay. AC is the native dinucleotide core sequence. Dots show each transfection replicate, bars show the mean, error = s.d. n=3 transfection replicates in HEK293FT cells. P-value determined by one-tailed t-test. **G.** Flow cytometry measuring mCherry$^+$ cells 18 days

after LSR and donor co-electroporation into WT K562 cells that lack a landing pad. attD donor contains an EF-1α promoter driving mCherry expression and attD donor transfected with a non-matching LSR is a negative control (n=2 transfection replicates). **H.** Schematic of optimized integration site mapping assay, a modified version of UDiTaS (Giannoukos et al., 2018). Addition of a round of amplification using a nested donor primer is expected to enrich for desired target-derived reads, which includes both donor-only reads and donor-genome junction reads (see Methods for details). An additional, optional, UMI can be provided in the donor proximal to the donor-genome junction to map unique integrations. **I.** Proportion of reads derived from different sources in the integration site mapping assay. The proportion of reads from each source before assay optimization on the left, and after optimization on the right. Both runs are Cp36 circular donor experiments, but in two different cell types (HEK293FT on the left, K562 on the right). Target-derived reads are those that come from the donor only (light green) or the donor-genome integration junction reads (dark green). **J.** Genome-wide integration site mapping by next generation sequencing to measure the percentage of reads found in the genome outside the expected landing pad. For Kp03, Ec03, n = 2 independent clonal landing pad lines were used, and for Pa01 n = 3 clonal landing pad lines were used, with maximal mCherry 11 days post donor electroporation. For Bxb1, three technical replicates (starting from different gDNA aliquots) of a single clonal landing pad line with maximal mCherry 11 days post donor electroporation are shown. Raw (non-unique) reads found at off-targets as a percentage of all reads are shown (* = $P < 0.05$, one-tailed t-test; Bxb1/Kp03 $P = 0.0001$; Pa01/Kp03 $P = 0.0002$; Ec03/Kp03 $P = 0.02$). Numbers near the top of each bar indicate the total number of off-target loci on the left, and below in parentheses are the subset of those sites that replicate in landing pad cell lines (left) and the subset that replicate in wild-type cell lines (right). **K.** Target site motifs generated from human genome off-target sites that were found to be reproducible across biological replicate experiments. The sequence motif for reproducible Kp03 off-target sites (730 sites in total) is shown on top, and the sequence motif for reproducible Pa01 (16 sites in total) is

shown on the bottom. In total, 8 Kp03 biological replicate experiments and 7 Pa01 biological replicate experiments were performed. Core dinucleotides are strongly conserved among integration sites for both candidates. The attB sequence, as determined from the LSR database, is shown in black text. **L.** Flow cytometry measuring $mCherry^{+}$ cells 18 days after LSR and donor co-electroporation into WT K562 cells that lack a landing pad. The donor plasmid contains an EF-1α promoter driving mCherry expression and attD donor transfected with a non-matching LSR is the negative control. (n = 2 transfection replicates)

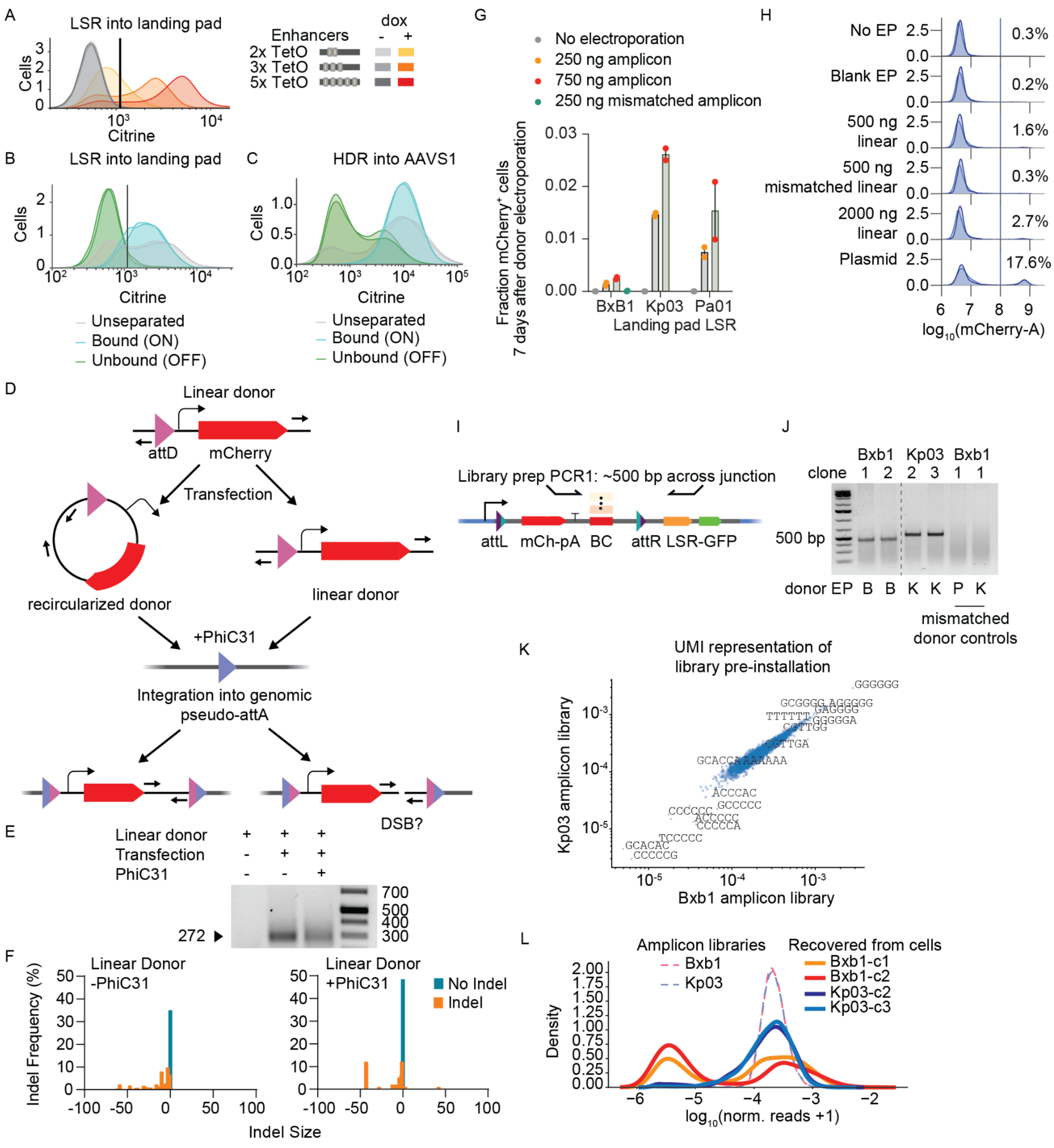

Supplementary Figure 3: Parallel reporter assay using magnetic separation and linear DNA library installation in landing pads.

A. Flow cytometry measurements of the landing pad citrine reporter 2 days after induction with doxycycline, in a distinct replicate from the experiment shown in **Fig. 3B**. This replicate is

time-matched with the LSR landing pad PRA shown in **Fig. 3C**, wherein reporters were delivered, selected with puromycin for 8 days, grown for 2 weeks, and then induced with doxycycline for 2 days before analysis. Vertical line marks the linear gate for Citrine$^+$ cells, which are shown in **Fig. 3C** (n=1 cell line replicate). **B.** Magnetic separation for LSR landing pad PRA cells, corresponding to the samples sequenced and shown in **Fig. 3C**. Unseparated sample is the pooled, dox-induced cells before mixing with magnetic beads. There are two colored distributions corresponding to two biological replicates for each separation fraction. **C.** Magnetic separation for HDR-integrated PRA cells, corresponding to the samples sequenced and shown in **Fig. 3C**. **D.** Schematic of potential mechanisms of linear donor integration. Upon transfection, the linear donor may either recircularize and get integrated as a circular donor into the attA, or remain linear, leading to double stranded breaks upon integration. Arrows indicate PCR primers used to test these mechanisms. **E.** Gel electrophoresis of PCR products using the primer pair illustrated in **D** on the untransfected and transfected PhiC31 attD linear donor amplicon, with and without PhiC31. Expected size of the PCR amplicon upon rejoining of the two donor ends without insertions or deletions is 272 bp. Uncropped gel shown at end of Supplementary Information File. **F.** Indel size distribution frequency of the PCR products shown in (**E**)**. G.** Efficiency of amplicon library integration measured by flow cytometry to determine the fraction of mCherry$^+$ cells 7 days after amplicon electroporation. Each condition, except the mismatched control, was tested in two different clonal landing pad lines, shown as dots (n=2, bar=mean, error=s.e.m.). The mismatched control is Kp03 or Pa01 amplicon electroporated into the same Bxb1 clonal landing pad line. **H.** Efficiency of linear donor integration with other doses, in Kp03 landing pad cells. The mismatch control uses the Bxb1 amplicon donor in Kp03 landing pad cells. 4615 ng of plasmid donor was used to provide an equimolar dose as 2000 ng of amplicon donor. Each donor condition was tested in two different clonal Kp03 landing pad lines, shown as differently shaded histograms. The gate for mCherry is shown as a vertical line and the average percentage of mCherry$^+$ cells is indicated. **I.** Schematic of junction PCR to detect the 3′

donor-genome junction upon integration of a 1.2 kb attP-mCherry-pA-Barcode amplicon into the landing pad cells. **J.** Gel electrophoresis of junction PCR from template gDNA harvested 8 days after electroporation with 750 ng of amplicon. Mismatched donor controls were Bxb1 landing pad cells electroporated with the Pa01 (lane 7, "P") or Kp03 (lane 8, "K") amplicon donor. The product of this junction PCR was used as input to two rounds of library preparation PCR to add adapters and indices for sequencing the barcodes. Uncropped gel shown at end of Supplementary Information File. **K.** Comparison of normalized read counts for the pre-installation amplicon libraries for Bxb1 and Kp03. Some of the 6xN randomized barcodes are labeled. **L.** Distribution of barcode read counts from amplicon libraries before and after integration in cells. A pseudocount was added to each element, before normalization by total sample counts.

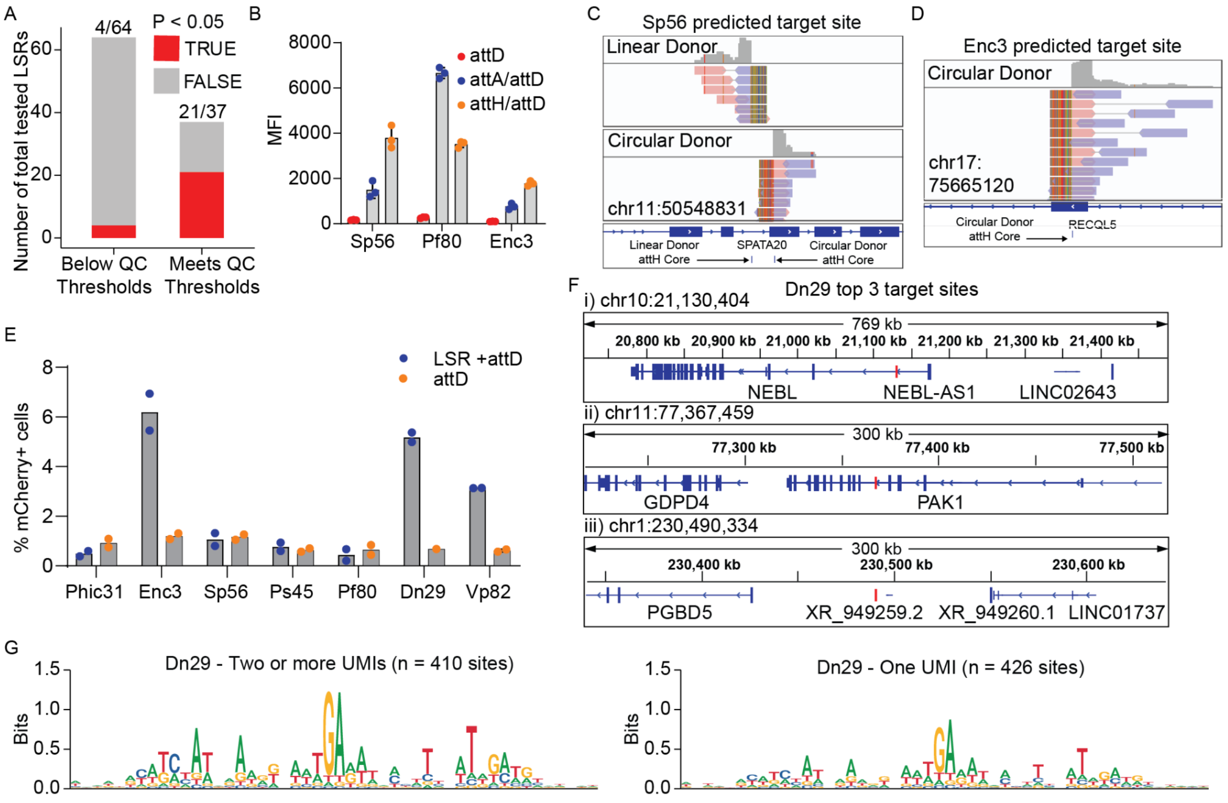

Supplementary Figure 4. Human genome-targeting recombinases target specific and predictable sites.

A. Proportion of genome-targeting LSR candidates that mediate significant recombination in the plasmid recombination assay with and without application of quality control (QC) thresholds for LSR candidate selection. The numbers above each bar indicate the (number of candidates that met $P < 0.05$ in the plasmid recombination assay) / (total number of tested candidates). **B.** Plasmid recombination assay for top genome-targeting candidates using predicted attH sites (bars show the mean, error = s.d. n=3 transfection replicates). **C.** Reads at top integration site for Sp56. Reads that align in the forward direction are shown in red and those aligning in the reverse direction are shown in blue, with a gray line connecting paired reads. The orientation and location of the integration changes when using a linear donor, whereas the exact predicted integration site is targeted with a circular donor. **D.** Same as (**C**), but for the predicted target site

of Enc3. **E.** Human genome integration efficiency assay results of the top candidates. PhiC31 is a previously known genome targeting LSR used as a control, although its efficiency is below the limit of detection (~1% of cells). Bars are mean, dots are individual transfections. n=2 electroporation replicates. **F.** The top three integration sites for Dn29, shown in their genomic context. The red line indicates the exact position of integration, with introns and exons of nearby genes in blue. **G.** A comparison of sequence motifs built from integration sites with two or more detected UMIs across biological replicates (left), or those sites with only one UMI (right).

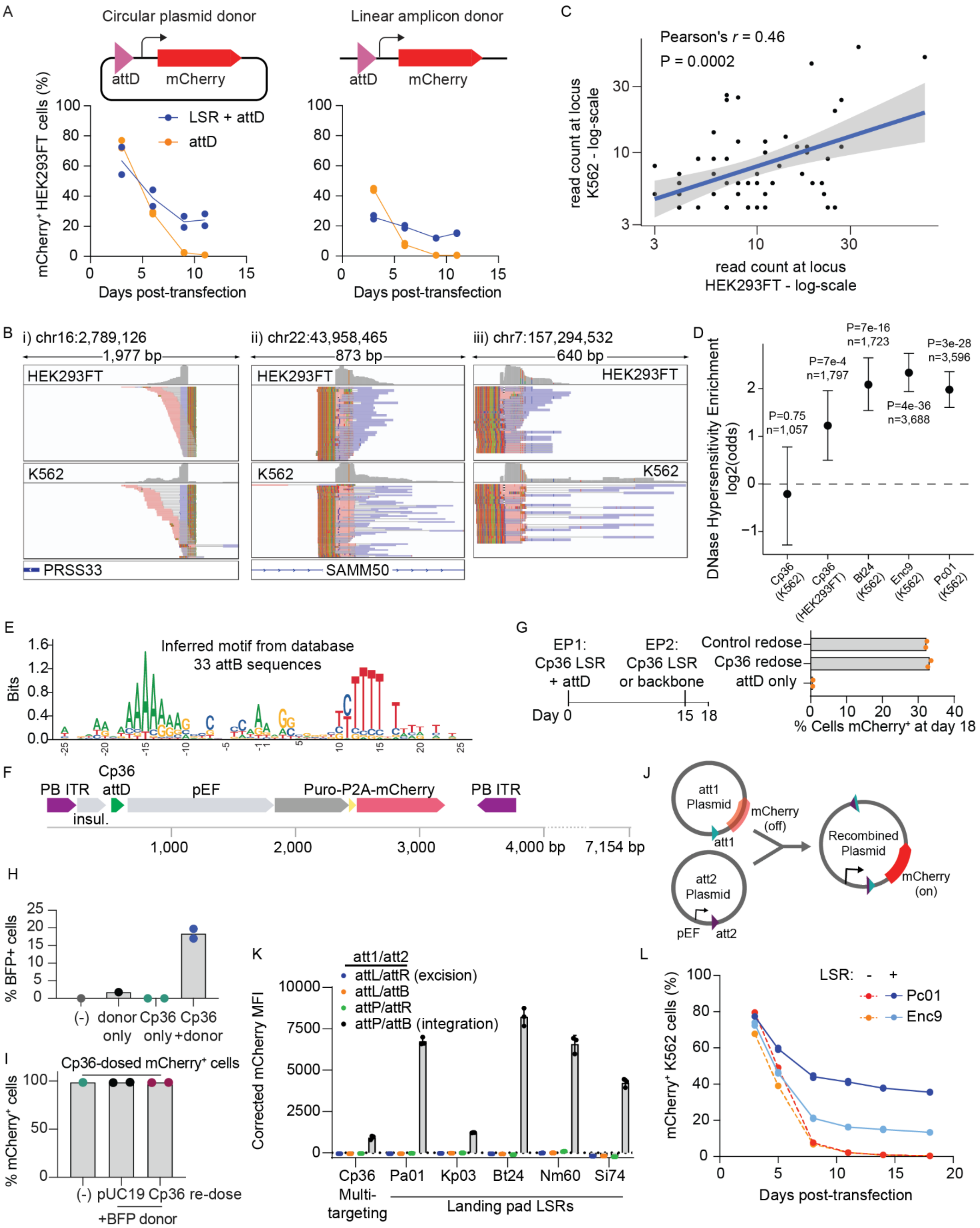
A
Circular plasmid donor
Linear amplicon donor
attD
mCherry
LSR + attD
attD
mCherry⁺ HEK293FT cells (%)
Days post-transfection
C
Pearson's r = 0.46
P = 0.0002
read count at locus K562 - log-scale
read count at locus HEK293FT - log-scale
B
i) chr16:2,789,126
1,977 bp
ii) chr22:43,958,465
873 bp
iii) chr7:157,294,532
640 bp
HEK293FT
K562
PRSS33
SAMM50
D
DNase Hypersensitivity Enrichment log2(odds)
P=0.75 n=1,057
P=7e-4 n=1,797
P=7e-16 n=1,723
P=4e-36 n=3,688
P=3e-28 n=3,596
Cp36 (K562)
Cp36 (HEK293FT)
Bt24 (K562)
Enc9 (K562)
Pc01 (K562)
E
Inferred motif from database
33 attB sequences
Bits
G
EP1: Cp36 LSR + attD
EP2: Cp36 LSR or backbone
Day 0
15 18
Control redose
Cp36 redose
attD only
% Cells mCherry⁺ at day 18
F
PB ITR
Cp36 attD
insul.
pEF
Puro-P2A-mCherry
PB ITR
1,000
2,000
3,000
4,000 bp
7,154 bp
J
att1 Plasmid
mCherry (off)
att1
att2 Plasmid
pEF
att2
Recombined Plasmid
mCherry (on)
H
% BFP+ cells
(-)
donor only
Cp36 only
Cp36 +donor
I
Cp36-dosed mCherry⁺ cells
% mCherry⁺ cells
(-)
pUC19
Cp36 re-dose
+BFP donor
K
att1/att2
attL/attR (excision)
attL/attB
attP/attR
attP/attB (integration)
Corrected mCherry MFI
Cp36 Multi-targeting
Pa01
Kp03
Bt24
Nm60
Si74
Landing pad LSRs
L
LSR: - +
Pc01
Enc9
mCherry⁺ K562 cells (%)
Days post-transfection

Supplementary Figure 5. Multi-targeting recombinases are efficient and unidirectional integrases.

A. Cp36 co-transfected with either a circular plasmid or linear amplicon attD-pEF-1α-mCherry donor in HEK293FT cells, compared with attD-only control, as measured by flow cytometry. Line shows the mean. (n = 2 transfection replicates) **B.** Integration site mapping shows precise integration into the same sites in multiple genomic DNA fragments from cells post-transfection with Cp36, suggesting these are recurring hotspots. Aligned reads colored according to forward strand (red) or reverse strand (blue), with paired reads joined by a black line. Soft-clipped portions of the aligned reads are colored to show where the read crosses over from the human genome into the Cp36 donor sequence, and the reference genome sequence on the bottom. The top three integration sites specified in **Fig. 5C** are shown. **C.** Correlation between read counts from the Cp36 integration site mapping assay across HEK293FT and K562 cell lines. The top 61 shared loci, all of which are found among the top 200 most frequently targeted sites in the two cell types are shown. Two-sided Pearson's test for correlation between paired samples used to estimate statistical significance. The blue line indicates the linear regression estimated fit, the gray band indicates the 95% confidence interval. **D.** Enrichment of target sites in DNase hypersensitivity peaks for several multi-targeters. Two-sided Fisher's exact test was used to calculate statistical significance of each enrichment. Center points indicate estimated $\log_2$(odds ratio) for each test. Nominal (uncorrected) P-values and number of relevant integration sites are shown above each relevant lane. Error bars indicate the 95% confidence interval. **E.** Target site motif as predicted using 33 attB sequences in the LSR-attachment site database that are targeted by LSRs that fall in the same 50% amino acid identity cluster as Cp36. Method used to construct this motif is the same as in **Fig. 1H** and **Supplementary Fig. 1E**. **F.** Schematic of donor plasmid used for direct comparison of Cp36 and PiggyBac (PB) that contains both the PB inverted terminal repeats (ITRs) and the Cp36 attD. **G.** Schematic on the

left depicts a Cp36 re-dosing experiment wherein Cp36 and an mCherry donor are used to generate mCherry$^+$ cells, and then Cp36 enzyme or the empty LSR expression backbone is re-dosed, followed by flow cytometry to measure excision of the mCherry cargo. On the right, percentage of mCherry$^+$ cells on day 18 as measured by flow cytometry (n=2 transfection replicates). **H.** Integration of the BFP donor by Cp36. K562 cells were electroporated with 2400 ng of Cp36 plasmid and 3000 ng of BFP donor plasmid and BFP was measured by flow cytometry after 12 days. Dash refers to unelectroporated cells, and the Cp36- or donor-only conditions include pUC19 stuffer plasmid so the mass delivered is equal. **I.** Cp36-dosed mCherry$^+$ and puromycin-selected cells were analyzed by flow cytometry 13 days post-electroporation with 2000 ng of BFP donor and an equimolar dose, 1600 ng, of Cp36 plasmid (or pUC19 stuffer plasmid). Dash shows unelectroporated control, n=2 electroporation replicates. **J.** Schematic of plasmid recombination assay to determine directionality of LSR recombination. In addition to the original attP/attB, attL/attR were also used in various combinations with attP/attB. If significant mCherry$^+$ fluorescence is detected in the attL/attR experiment, it would imply that the LSR was bi-directional, being capable of excision. The LSR is co-transfected on a third plasmid. **K.** Plasmid recombination assay to determine LSR directionality. mCherry MFI is corrected according to a control that lacked the LSR plasmid. Bars are mean, dots are transfection replicates. (error=s.d. n=3 transfection replicates) **L.** Additional multi-targeting LSR candidates validated using the pseudosite integration assay. The two additional candidates, Pc01 and Enc9, are considered multi-targeting, with Pc01 containing DUF4368 and residing in a clade that is closely related to the primary multi-targeting clade, and Enc9 residing directly in the primary multi-targeting clade shown in **Fig. 1B** and **Supplementary Fig. 1A**. Line shows the mean. (n=2 electroporation replicates of 500ng donor and 600ng LSR plasmid).

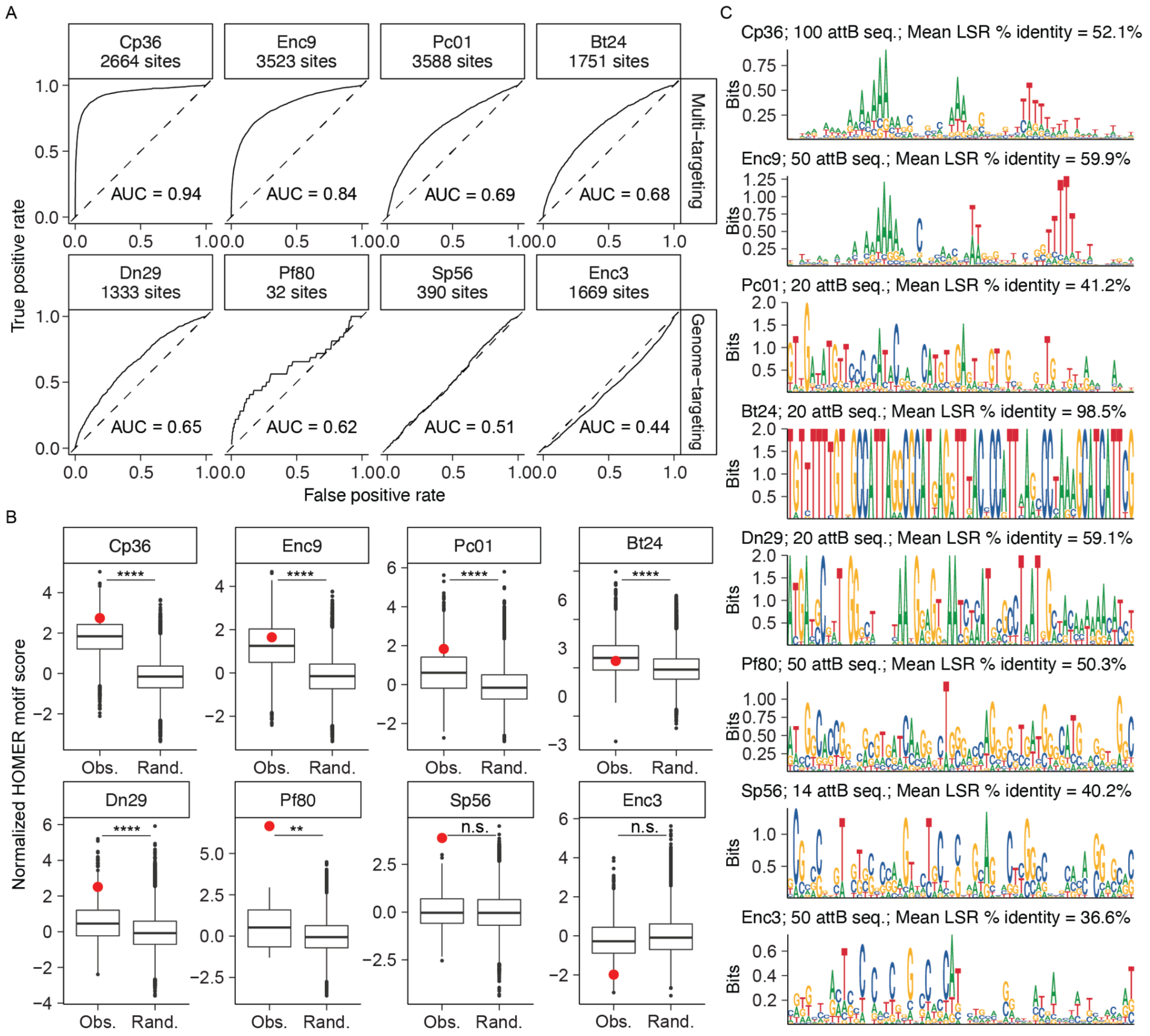

Supplementary Figure 6. *Post hoc* identification of human genome integration sites using database sequence motifs.

A. Performance of database-derived sequence motifs to predict human genome integration sites as measured by ROC curve analysis. Sequence motifs for each LSR were automatically generated from the bacterial sequence database by selecting non-redundant (95% nucleotide identity) attB sequences of related LSR orthologs. These motifs were then searched against true integration sites and randomly selected background sequences using the HOMER motif

analysis software (Heinz et al., 2010). ROC curves were generated by sliding across a relevant range of motif score cutoffs and calculating the false positive rate (x-axis) and true positive rate (y-axis) at each cutoff. The area under the curve (AUC) was then calculated as a single measure of predictive performance. Each ROC curve is labeled with the relevant LSR name and the number of integration sites detected across all relevant experiments. **B.** Comparing distributions of normalized HOMER motif scores in experimentally observed integration sites ("Obs.") vs. randomly selected background sequences ("Rand.") depicted in boxplots with median, 1st and 3rd quartiles, 1.5 x IQR as whiskers, and outliers as points. One-sided Wilcoxon rank-sum test for significant differences between groups (** is $P < 0.01$, **** is $P < 0.0001$, n.s. is not significant; 28,315 random background sequences used for all tests; Cp36, n = 2,664 obs. sites, $P < 2.2e-16$; Enc9, n = 3,523 obs. sites, $P < 2.2e-16$; Pc01, n = 3,588 obs. sites, $P < 2.2e-16$; Bt24, n = 1,751 obs. sites, $P < 2.2e-16$; Dn29, n = 1,333 obs. sites, $P < 2.2e-16$; Pf80, n = 32 obs. sites, $P = 0.008$; Sp56, n = 390 obs. sites, $P = 0.2$; Enc3, n = 1,669 obs. sites, $P = 1.0$;). Red points indicate the normalized HOMER motif score for the observed integration site with the most experimentally detected integration events relative to all other integration sites for each LSR. **C.** Final sequence motifs used to predict human genome integration sites for each LSR. Each sequence is labeled with the corresponding LSR, the number of attB sequences used to build the motif, and the mean percentage amino acid identity of all the LSR orthologs that were used to identify related attB sequences.

Supplementary Note 1. Further discussion of the biological role of LSR target genes.

We reasoned that any enrichment of the genes that were targeted and therefore disrupted upon LSR-mediated MGE integration could indicate an evolved strategy for LSR-carrying MGEs. We

identified Pfam domains that were enriched among target genes (**Supplementary Fig. 1B**). Enriched domains were found in Magnesium chelatases, Competence proteins, Type II/IV secretion system proteins, and HNH endonucleases, among others. Next, we performed gene ontology (GO) pathway analysis of the target genes, and identified six pathways that were significantly enriched (FDR < 0.1; **Supplementary Fig. 1C**). Notably, the GO term "establishment of competence for transformation" (GO:0030420) was the most significantly enriched pathway with 15 target gene clusters being annotated with this term. Among these target genes was the ComK transcription factor and other ComG operon proteins, suggesting that disrupting competence and DNA transformation is a common strategy for LSR-carrying MGEs. Reasoning that LSRs may have also evolved to target host anti-phage defense systems upon integration, we annotated relevant genomes using DefenseFinder (Abby et al., 2014; Tesson et al., 2021), and we searched for LSR target genes that occurred in or near these identified systems. We identified some defense genes that were targeted by integrases, including CRISPR spacer acquisition gene *cas2*, CRISPR helicase/nuclease *cas3*, Type I restriction modification enzymes, Hachiman defense gene *hamA*, and a UvrD-like helicase gene. However, defense genes were rarely targeted by LSRs, and we did not see any enrichment of target genes near defense genes, suggesting this is not a common strategy (**Supplementary Fig. 1D**). These findings support an evolved strategy adopted by LSR-carrying MGEs that limits further horizontal gene transfer primarily through disruption of competence.

Supplementary Note 2. Discussion of *post hoc* identification of human genome integration sites using database sequence motifs.

We reasoned that in addition to a BLAST-based search used to identify the genome-targeting candidates presented in **Fig. 4**, it may also be possible to search the human genome using a sequence motif that was derived from natural attB sequences. We decided to perform a *post hoc* analysis of the genome-targeting and multi-targeting candidates in this study to determine

how feasible a motif-based search would be. Starting with each experimentally characterized candidate, we built sequence motifs by iteratively adding natural attB sequences of the next most closely related LSR ortholog, only adding additional attB sequences if they were 95% identical or less to already selected attB sequences. We built motifs of 20, 50 and 100 such attB sequences. Then these motifs were searched against the experimentally observed human integration sites, and approximately 30,000 randomly selected human genome sequences. Next, we iterated across motif score cutoffs and calculated the true positive rate and the false positive rate at each cutoff, generating a ROC curve (**Supplementary Fig. 6A**). For each LSR, the motif with the greatest AUC was selected.

We found that the sequence motifs belonging to the multi-targeting candidates performed quite well, with AUC values ranging from 0.94 for the Cp36 motif to 0.68 for the Bt24 motif. For the genome-targeting candidates the performance of the sequence motifs varied, ranging in AUC values from 0.65 for Dn29 to 0.44 for Enc3. All of these motifs assigned significantly higher scores to observed integration sites than randomly selected controls, except for Sp56 and Enc3, which did not differ significantly (Wilcoxon rank-sum test; $P < 0.0001$ for Cp36, Enc9, Pc01, Bt24, and Dn29, $P < 0.01$ for Pf80, $P > 0.05$ for Sp56 and Enc3). Despite the relatively poor performance of the Pf80 motif and the Sp56 motif, they did assign the highest motif scores to the most frequently targeted human genome integration sites, suggesting that there is predictive value to their database-derived sequence motifs (**Supplementary Fig. 6B**). Upon visual inspection of the motifs we see a variety of patterns, with Cp36 and Enc9 motifs having the characteristic AT rich motifs typical of many multi-targeting LSRs, and others such as Dn29 and Bt24 having less variation and less well-defined boundaries (**Supplementary Fig. 6C**).

These results suggest that there is value in taking a motif-based sequence search when prioritizing multi-targeting and genome-targeting candidates. The potential targeting profile of

multi-targeters could be better understood prior to experimental validation, as with Cp36 and Enc9, and genome-targeting candidates could be selected based on those that have high, outlier motif matches that could indicate higher specificity, such as for Pf80. We hypothesize that the difference in performance between motifs may be explained by the different selection pressures placed on multi-targeting and single-targeting LSRs, where multi-targeting LSRs are more likely to maintain their relaxed sequence specificity across larger evolutionary distances due to a greater abundance of possible target sites, leading to more accurate sequence motifs. These results could also have been influenced by the efficiency of the LSR in human cells or epigenetic modifications at the target site such as those that influence chromatin accessibility (**Supplementary Fig. 5D**). We expect that as publicly-available sequence databases grow in size and more LSR-attachment site pairs are identified, the quality of the motif-based predictions will increase due to improved coverage of the target sequences for any given LSR.

Supplementary Figure 3E uncropped gel

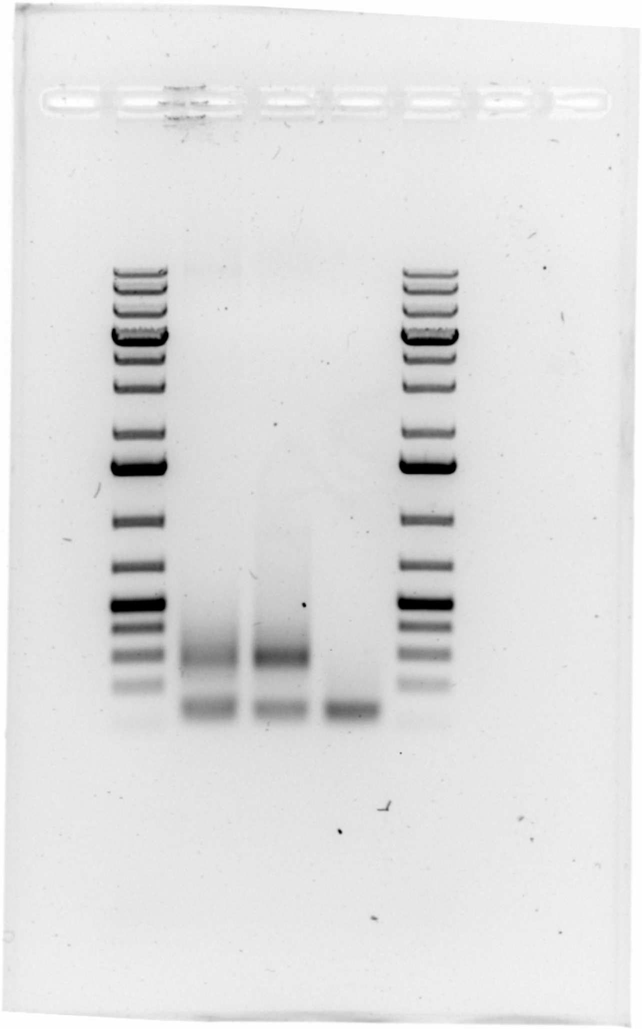

Supplementary Figure 3J uncropped gel

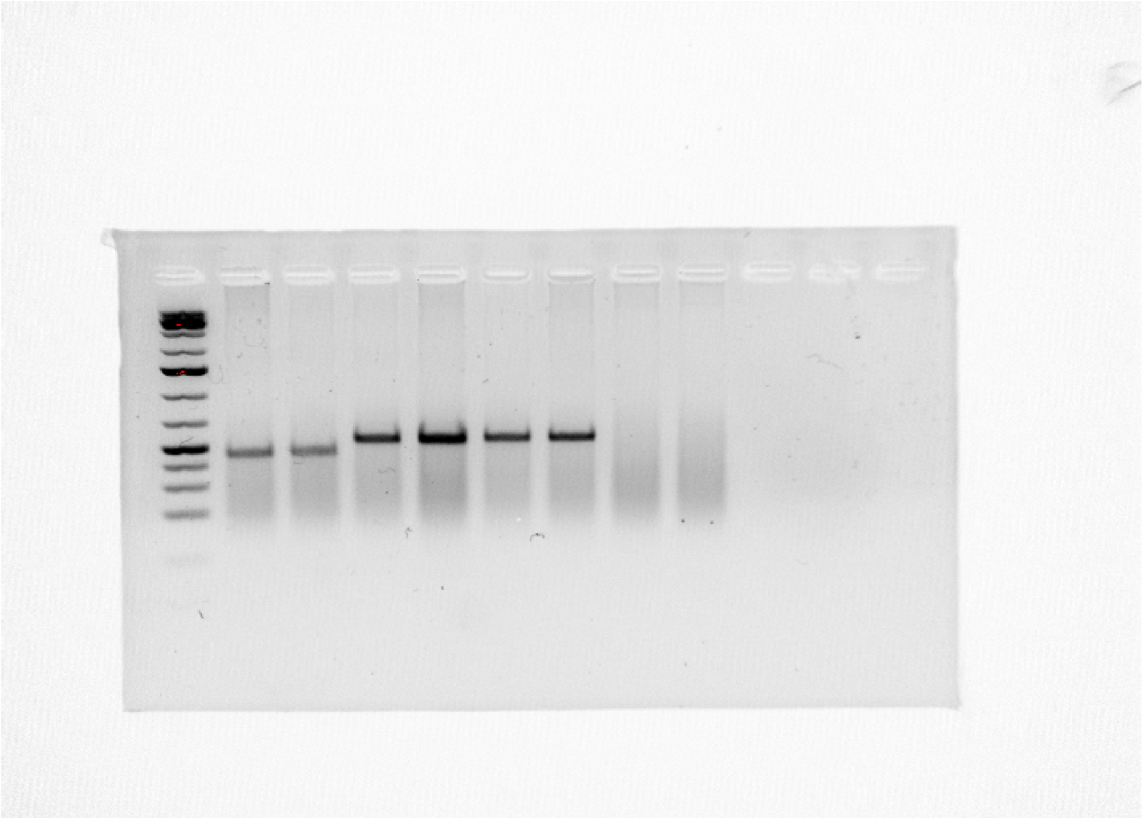

References

Abby, S. S., Néron, B., Ménager, H., Touchon, M., & Rocha, E. P. C. (2014). MacSyFinder: a program to mine genomes for molecular systems with an application to CRISPR-Cas systems. *PloS One*, *9*(10), e110726.

Giannoukos, G., Ciulla, D. M., Marco, E., Abdulkerim, H. S., Barrera, L. A., Bothmer, A., Dhanapal, V., Gloskowski, S. W., Jayaram, H., Maeder, M. L., Skor, M. N., Wang, T., Myer, V. E., & Wilson, C. J. (2018). UDiTaS^{TM}, a genome editing detection method for indels and genome rearrangements. *BMC Genomics*, *19*(1), 212.

Heinz, S., Benner, C., Spann, N., Bertolino, E., Lin, Y. C., Laslo, P., Cheng, J. X., Murre, C., Singh, H., & Glass, C. K. (2010). Simple combinations of lineage-determining transcription factors prime cis-regulatory elements required for macrophage and B cell identities.

Molecular Cell, *38*(4), 576–589.

Tesson, F., Hervé, A., Touchon, M., d'Humières, C., Cury, J., & Bernheim, A. (2021). Systematic and quantitative view of the antiviral arsenal of prokaryotes. In *bioRxiv*. https://doi.org/10.1101/2021.09.02.458658
