# Supplementary material for: Systematic discovery of recombinases for efficient integration of large DNA sequences into the human genome
Source: Nat Biotechnol. 2022 Oct 10;41(4):488–99. doi: 10.1038/s41587-022-01494-w (PMC10083194; doi:10.1038/s41587-022-01494-w)
Supplement: Supplementary file 2 — Reporting Summary [file 41587_2022_1494_MOESM2_ESM.pdf]

nature portfolio

Corresponding author(s): Patrick Hsu, Ami Bhatt, Michael Bassik, Lacramioara Bintu

Last updated by author(s): 06/13/2022

Reporting Summary

Nature Portfolio wishes to improve the reproducibility of the work that we publish. This form provides structure for consistency and transparency in reporting. For further information on Nature Portfolio policies, see our Editorial Policies and the Editorial Policy Checklist.

Statistics

For all statistical analyses, confirm that the following items are present in the figure legend, table legend, main text, or Methods section.

n/a	Confirmed	
☐	☒	The exact sample size (*n*) for each experimental group/condition, given as a discrete number and unit of measurement
☐	☒	A statement on whether measurements were taken from distinct samples or whether the same sample was measured repeatedly
☐	☒	The statistical test(s) used AND whether they are one- or two-sided<br>*Only common tests should be described solely by name; describe more complex techniques in the Methods section.*
☐	☒	A description of all covariates tested
☐	☒	A description of any assumptions or corrections, such as tests of normality and adjustment for multiple comparisons
☐	☒	A full description of the statistical parameters including central tendency (e.g. means) or other basic estimates (e.g. regression coefficient) AND variation (e.g. standard deviation) or associated estimates of uncertainty (e.g. confidence intervals)
☐	☒	For null hypothesis testing, the test statistic (e.g. *F*, *t*, *r*) with confidence intervals, effect sizes, degrees of freedom and *P* value noted<br>*Give P values as exact values whenever suitable.*
☐	☒	For Bayesian analysis, information on the choice of priors and Markov chain Monte Carlo settings
☐	☒	For hierarchical and complex designs, identification of the appropriate level for tests and full reporting of outcomes
☐	☒	Estimates of effect sizes (e.g. Cohen's *d*, Pearson's *r*), indicating how they were calculated

Our web collection on statistics for biologists contains articles on many of the points above.

Software and code

Policy information about availability of computer code

Data collection	Attune NxT Flow cytometer Software v5.1.1, Illumina MiSeq Control Software v4.0.0.1769, BD Accuri C6 Software v227, Biorad ZE5 Cell Analyzer Everest Software v3.1
Data analysis	Microsoft Excel v16.54, Flowjo v10.7.1, GraphPad Prism v9.3.1, TaxonKit v0.7.1, Prodigal v2.6.3, HMMER v3.3.2, MGEfinder v1.0.6, PhyloPhlAn v3.0.2, MMseqs2 v13-45111, MAFFT v7.471, IQ-TREE v2.1.2, R 4.1.0, ggseqlogo v0.1, BWA MEM v0.7.17, Biopython v179, CytoFlow v1.0, bcl2fastq Conversion Software v2.20, HT-recruit-Analyze (original version), VirSorter2 v2.2.3, ConjScan v1.0.2, ICEBerg v2.0, BLAST v2.12.0, InterProScan v1.8.0_152, DefenseFinder v1.0.8, CRISPResso2 v2.0.20b

For manuscripts utilizing custom algorithms or software that are central to the research but not yet described in published literature, software must be made available to editors and reviewers. We strongly encourage code deposition in a community repository (e.g. GitHub). See the Nature Portfolio guidelines for submitting code & software for further information.

Data

Policy information about availability of data

All manuscripts must include a data availability statement. This statement should provide the following information, where applicable:

- Accession codes, unique identifiers, or web links for publicly available datasets
- A description of any restrictions on data availability
- For clinical datasets or third party data, please ensure that the statement adheres to our policy

Publicly-available RefSeq and Genbank genomes were used to generate the LSR database. Data to support the results are in the main text and the Supplementary Information. Illumina sequencing datasets generated in this study are available on NCBI Sequence Read Archive, BioProject PRJNA778877. Additional data will be made available upon reasonable request.

Field-specific reporting

Please select the one below that is the best fit for your research. If you are not sure, read the appropriate sections before making your selection.

☒ Life sciences ☐ Behavioural & social sciences ☐ Ecological, evolutionary & environmental sciences

For a reference copy of the document with all sections, see nature.com/documents/nr-reporting-summary-flat.pdf

Life sciences study design

All studies must disclose on these points even when the disclosure is negative.

Sample size	The experiments described in this study were done for the first time. No pre-specified effect size could be determined a priori.<br>For MS, in general, two replicates are acceptable if the overlap between them is good (e.g. r^2 greater than or equal to 0.80). In this study we used a minimum of two replicates, with excellent reproducibility between replicates.
Data exclusions	No data were excluded from the study.
Replication	For plasmid recombination assay, 3 replicates were performed per transfection. For lentiviral genome integration assay, two polyclonal cell lines and up to 4 clonal cell lines were tested per recombinase. For integration site mapping, two biological replicates included per sample. All replicate values are indicated in figure legends. Mini PRA data performed in 2 cell line replicates. All data supports replicability.
Randomization	Randomization was not relevant for this study. The same cell lines were used for positive and negative controls per experiment, which does not require allocating samples into experimental groups.
Blinding	Investigators were not blinded, we are a small team performing cell & molecular biology experiments.

Reporting for specific materials, systems and methods

We require information from authors about some types of materials, experimental systems and methods used in many studies. Here, indicate whether each material, system or method listed is relevant to your study. If you are not sure if a list item applies to your research, read the appropriate section before selecting a response.

Materials & experimental systems

n/a	Involved in the study	
☒	☐	Antibodies
☐	☒	Eukaryotic cell lines
☒	☐	Palaeontology and archaeology
☒	☐	Animals and other organisms
☒	☐	Human research participants
☒	☐	Clinical data
☒	☐	Dual use research of concern

Methods

n/a	Involved in the study	
☒	☐	ChIP-seq
☐	☒	Flow cytometry
☒	☐	MRI-based neuroimaging

Eukaryotic cell lines

Policy information about cell lines

Cell line source(s)	HEK293FT from Thermo Fisher, K562 and HEK293T from ATCC , LentiX from Takara Bio.
Authentication	None of the cell lines used were authenticated.
Mycoplasma contamination	Cell lines not tested for mycoplasma.
Commonly misidentified lines<br>(See ICLAC register)	No commonly misidentified cell lines were used.

Flow Cytometry

Plots

Confirm that:

☒ The axis labels state the marker and fluorochrome used (e.g. CD4-FITC).

☒ The axis scales are clearly visible. Include numbers along axes only for bottom left plot of group (a 'group' is an analysis of identical markers).

☒ All plots are contour plots with outliers or pseudocolor plots.

☒ A numerical value for number of cells or percentage (with statistics) is provided.

Methodology

Sample preparation	HEK293FT cells were dissociated with TrypLE and resuspended in Stain Buffer (BD Biosciences). 100uL of K562 cells were resuspended in Stain buffer.
Instrument	Attune NxT Flow Cytometer, BD Accuri C6 Cytometer, BioRad ZE5 Cytometer
Software	Flowjo v10.7.1, CytoFlow v1.0
Cell population abundance	Since samples are single cell lines, relevant population is the entire sample excluding dead cells and doublets, often >60% of the sample
Gating strategy	Cells initially gated on FSC/SSC for single cells to exclude doublets and dead cells. GFP and mCherry gates determined by gating on cells transfected with an empty plasmid backbone (pUC19).

☒ Tick this box to confirm that a figure exemplifying the gating strategy is provided in the Supplementary Information.
